# The Influence of Focus Marking on Pronoun Resolution in Dialogue Context

**DOI:** 10.3389/fpsyg.2021.684639

**Published:** 2021-07-26

**Authors:** Liam P. Blything, Juhani Järvikivi, Abigail G. Toth, Anja Arnhold

**Affiliations:** ^1^Department of Linguistics, University of Alberta, Edmonton, AB, Canada; ^2^Department of Psychology, University of Liverpool, Liverpool, United Kingdom; ^3^Department of Artificial Intelligence, University of Groningen, Groningen, Netherlands

**Keywords:** focusing, prosody, cleft sentences, pronouns, eye-tracking, individual differences

## Abstract

Using visual world eye-tracking, we examined whether adults (*N* = 58) and children (*N* = 37; 3;1–6;3) use linguistic focussing devices to help resolve ambiguous pronouns. Participants listened to English dialogues about potential referents of an ambiguous pronoun he. Four conditions provided prosodic focus marking to the grammatical subject or to the object, which were either additionally it-clefted or not. A reference condition focussed neither the subject nor object. Adult online data revealed that linguistic focussing via prosodic marking enhanced subject preference, and overrode it in the case of object focus, regardless of the presence of clefts. Children’s processing was also influenced by prosodic marking; however, their performance across conditions showed some differences from adults, as well as a complex interaction with both their memory and language skills. Offline interpretations showed no effects of focus in either group, suggesting that while multiple cues are processed, subjecthood and first mention dominate the final interpretation in cases of conflict.

## Introduction

It is widely assumed that adult pronoun interpretation is guided by complex interactions of multiple cues (Arnold et al., 2000; Arnold, 2001, 2010; Järvikivi et al., 2005; Kaiser and Trueswell, 2008; Schumacher et al., 2017). A non-exhaustive list of factors that have been shown to influence adult pronoun resolution preferences includes grammatical role and/or order of mention (Keenan, 1976; Gernsbacher and Hargreaves, 1988; Järvikivi et al., 2005); verb semantics, semantic roles, connectives, coherence relations, or a combination thereof (Kehler et al., 2008; Pyykkönen and Järvikivi, 2010; Schumacher et al., 2017; Grüter et al., 2018); prosody (Maratsos, 1973); speaker eye-gaze/gesture (Goodrich Smith and Hudson Kam, 2012; Hawthorne et al., 2016); visual animacy (Cooper-Cunningham et al., 2020); language complexity/distance between the antecedent and pronoun (Hartshorne et al., 2015); as well as the presence/extent of dialogue, number of competitor referents, and focussing via questions (Serratrice and Allen, 2015). For example, adult processing studies have robustly demonstrated that personal pronouns are typically interpreted as co-referring with the grammatical subject and/or first mentioned entity of the preceding clause, i.e., the *giraffe* in (1) (Gernsbacher and Hargreaves, 1988; Crawley et al., 1990; Carreiras et al., 1995; Arnold et al., 2000; Järvikivi et al., 2005). Furthermore, children as young as 3-years-old also display subject and/or first mention preferences (Song and Fisher, 2005; Pyykkönen et al., 2010; Järvikivi et al., 2014; Hartshorne et al., 2015), although these effects appear to be weaker in magnitude and can often occur much later in the time course compared to adults (see Hartshorne et al., 2015, for a comprehensive review).

(1) The giraffe nudged the elephant beside the tree. He wanted to be friendly.

Different accounts have been proposed to explain this subject and/or first mention preference, for example in terms of probabilistic regularities of co-reference (Arnold, 2001, 2010) or discourse coherence (Kehler et al., 2008); hierarchies within grammatical relations (Keenan, 1976); and parallelism in the grammatical role of the pronoun and noun phrase referent (Sheldon, 1974; Smyth, 1994). Importantly, most accounts can be accommodated into a multiple constraints framework, which recognises that pronoun processing is sensitive to a wide range of linguistic and non-linguistic features (such as those listed earlier), and that some modulate or even override subject and first mention cues (Järvikivi et al., 2005; Kaiser and Trueswell, 2008; Schumacher et al., 2017). This means that previously reported effects of grammatical role and order of mention are at least partially a side product of their convergence with alternate cues that are often not incorporated into the experimental design. For example, in an SVO word order language like English, grammatical role and order of mention are most often confounded, with the subject entity being first mentioned and also occupying the semantic role of the agent. Thus, the giraffe in (1) simultaneously is the grammatical subject, first mention, and agent. Studies with flexible word order languages, such as German and Finnish, have allowed for the partial disentangling of these different cues, given that in an OVS word order the first-mentioned entity is instead aligned to the object and typically the patient role. These studies have shown that interpretative preferences appear most robust when grammatical role, order of mention, and semantic cues are aligned (SVO), but are significantly weakened when the cues are put into conflict (OVS), both for adults (Järvikivi et al., 2005; Kaiser and Trueswell, 2008; Schumacher et al., 2017) and even more clearly, for children (Blything et al., 2021). In fact, it has often been suggested that children differ from adults in terms of the ability to appropriately use (or suppress) multiple cues, which would hinder their ability to interpret the pronoun, though with a potential role for individual differences (Järvikivi et al., 2014; Hartshorne et al., 2015).

In the present study, we specifically follow-up on research addressing the role of discourse pragmatic linguistic focussing cues (Cowles et al., 2007; Foraker and McElree, 2007; Järvikivi et al., 2014; Colonna et al., 2015; Patterson et al., 2017) by investigating the effect of it-clefts and prosodic focus marking on pronoun resolution in adults and 3–6-year-old children. It-clefts and prosody are both linguistic means for marking information structure, i.e., how the utterance relates to the common ground of information shared between the speaker and the listener (Krifka, 2007, for an overview of the semantic and pragmatic literature on information structure). In other words, clefts and prosodic focus marking are employed by speakers to guide listeners’ attention and to explicitly signal how the speaker expects the listener to update their discourse model, i.e., their mental representation of the events and referents under discussion (Johnson-Laird, 1983; Zwaan and Radvansky, 1998). In particular, they mark the focus of an utterance, i.e., the new or contrastive information, separating it from the background or presupposed information (Krifka, 2007, for a discussion of more formal definitions), which has long been known to enhance processing speed and memory of the focused words (Cutler and Foss, 1977; Cutler and Fodor, 1979; Birch and Garnsey, 1995; Kember et al., 2019; Káldi and Babarczy, 2021). In turn, this memory advantage for the focussed element should serve pronoun interpretation because it is more available and therefore easier to establish as the pronoun referent. Indeed, processing of the pronoun is typically assumed to be determined by how easily the pronoun can be integrated with the character most foregrounded in the current mental representation (we return to this in our Discussion; also see Gundel et al., 1993; Zwaan and Radvansky, 1998).

It-cleft sentences mark the clefted entity as the focus of a presupposed event that is expressed within the clause (Chafe, 1976; Kiss, 1998; Lambrecht, 2001). Examples of both subject and object it-clefts can be seen in (2) and (3) respectively.

(2) It was the giraffe that nudged the elephant beside the tree. He wanted to be friendly.
(3) It was the elephant that the giraffe nudged beside the tree. He wanted to be friendly.

Various paradigms have investigated the effect of subject it-clefts in adult pronoun resolution. Some studies report that adults are more likely to attach personal pronouns to the subject antecedent when it is clefted than without clefting, suggesting that clefts have a unique influence over already robust cues that are inherently present like subjecthood, first mention and agentivity (Cowles et al., 2007; Foraker and McElree, 2007; Colonna et al., 2015). However, others have reported that adults show no difference in processing clefted compared to non-clefted subjects, suggesting that clefts do not show an influence over already robust cues (Colonna and Hemforth, 2014; Järvikivi et al., 2014; or marginal significance: Kaiser, 2011) or even that focussing reduces the subject preference (dubbed “anti-focus effect,” Colonna et al., 2012, 2015; de la Fuente, 2015; Patterson et al., 2017). Two of those studies (both using the visual word paradigm) also incorporated object it-clefts and revealed no significant reduction of general subject attachment preferences both online and offline (Kaiser, 2011; Järvikivi et al., 2014), while de la Fuente (2015) observed an anti-focus effect for object clefts using offline measures. Various differences in experimental design may be responsible for these conflicting findings, including whether or not the pronoun is preceded by a sentence boundary (cf., Colonna et al., 2012, 2015; Colonna and Hemforth, 2014; de la Fuente, 2015) or the fact that most studies lack a felicitous context that provides purpose for the it-cleft to contrastively focus a referent (with the exception of Kaiser, 2011; some of the studies in de la Fuente, 2015). For example, the majority of studies above presented an it-cleft after a sentence that simply introduced the characters, which does not align with the function that it-clefts serve in the real world; namely to contrastively focus something from prior discourse (Hornby, 1974). Thus, in the previous studies, the it-cleft more or less appeared “out of the blue.” The present study therefore employed a more felicitous context where the it-clefts follow a narrow focus non-contrastive question, such as *Who nudged the elephant beside the tree*?

Prosody is ubiquitous as a means of focus marking, especially in English, and often co-occurs with it-clefts in the real world. Interestingly, prosodic marking has been shown to be undiminished for clefts like (4) compared to non-cleft sentences with only prosodic marking like (5) (Arnhold, 2021). In fact, prosody seems to be an integral aspect of clefting. A cross-modal priming study by Cowles et al. (2007) reported that subject-clefts enhanced subject preference (measured by speed of naming its probe target word) only when the it-clefted entity was enhanced in its contrastive nature by prosodic marking.

(4) It was the GIRAFFE that nudged the elephant beside the tree. He wanted to be friendly.
(5) The GIRAFFE nudged the elephant beside the tree. He wanted to be friendly.

Importantly, prosodic cues may modulate interpretive preferences on their own—that is, in the absence of clefts. For example, Maratsos (1973) reported that prosodic focus marking modulated adult interpretive preferences of the pronoun *him*. When adults heard sentences like (6), most used the contrast signalled by the intonation on the pronoun to infer that the *giraffe* is now being nudged, rather than their interpretation under neutral prosodic marking that the *elephant* was once again affected by the action.

(6) The giraffe nudged the elephant beside the tree, and then the monkey nudged HIM.

Here, we will concentrate on the effect of prosodic focus marking not on the pronoun, but in the context preceding the pronoun, as in (5).

With regard to children, evidence suggests that sensitivity to prosodic focus marking starts to appear between 3 and 6 years of age, though children’s use of prosody in production and comprehension keeps developing for several years after that (Moore et al., 1993; Ito and Speer, 2008; Ito et al., 2014; Armstrong et al., 2016; overviews in Wells et al., 2004; Arnhold et al., 2016). Several studies have found that children use contrastive focus marking for predicting upcoming units of speech (Arnold, 2008; Ito et al., 2012, 2014; Sekerina and Trueswell, 2012), while Moore et al. (1993) showed that prosodic cues to (un)certainty (rising vs. falling intonation) modulate lexical cues (*I think* vs. *I know*). These studies are outside the domain of pronouns, but show that use of prosodic cues in comprehension tasks is developing within our age of interest, and can both enhance and hinder other language cues.

Sensitivity to clefting has been shown for children as young as 3–6 years of age (Ambridge et al., 2006; Theakston et al., 2014; Aravind et al., 2016). For example, in a study with 4-year-old German children, Järvikivi et al. (2014) found that children displayed an enhanced subject preference when a subject it-cleft was present; although their interpretive preferences were unaffected by the presence of an object it-cleft. Interestingly, in the same study, it-clefts (both subject and object) had no clear influence on adults’ pronoun interpretive preferences. The authors interpreted these findings as suggesting that children and adults may be sensitive to the same grammatical role and/or order of mention cues in reference resolution but that these constraints may not yet be fully acquired in children. However, as in most other studies, clefts in this study appeared without a preceding discourse context that rendered focus marking via clefting felicitous.

Prosodic effects may be another reason for the discrepancy of findings in previous studies investigating the influence of clefting on pronoun resolution for both children and adults. Almost none of the studies referenced above reported on the prosody of the spoken stimulus sentences used, which leaves a potentially important factor unaccounted for (the exception being Colonna et al., 2015, who used synthesised speech to control for prosody, but do not discuss its potential effects). Here, we expand on these previous studies by explicitly controlling and independently testing for the effects of prosody.

The present visual world paradigm investigated the role of linguistic focussing cues on adult and child real time (and offline) pronoun processing. This is the first pronoun study to date that has operationalised linguistic focussing cues as prosodic marking in the presence or absence of it-clefts, and in a discourse context that qualifies their contrastive function. Participants listened to spoken dialogues whilst their eye movements were tracked and time locked to the onset of the ambiguous pronoun *he*. Four of our five conditions used non-contrastive questions, where we manipulated whether the grammatical subject or object was prosodically marked as focussed and then either additionally it-clefted that referent or not (see Table 1). These four conditions are referred to as *subject focus-cleft absent, subject focus-cleft present, object focus-cleft absent*, and *object focus-cleft present*. We included a fifth condition (*broad focus*) to serve as a baseline, which did not focus either entity through a question or with linguistic focus cues. These conditions not only manipulated the focus, but also controlled other relevant aspects of information structure. In the *broad focus* condition, the entire test sentence constituted new information answering the preceding context question. In the other conditions, the preceding felicitous context question already established everything outside the focus as given information. Thus, for the subject focus conditions in Table 1, both speakers are aware that someone nudged the elephant before the crucial test sentence, whereas for the object focus conditions, it is established that the giraffe nudged someone. At the same time as establishing the division into focus vs. background and new vs. given, the preceding context also affects a third aspect of the information structure of the test sentence that is related and often correlated (including in our data), but can be separated both conceptually and empirically, namely the selection of a topic (cf. Krifka, 2007). Many scholars have identified the topic with given information (and the focus as new), but we here adopt the more precise definition of topic in terms of aboutness, following Reinhart (1981). Crucially, she concentrates on sentence topics, which have to correspond to an expression in the sentence, usually a noun phrase like *the elephant* or *the giraffe* (as opposed to discourse topics like *the events by the tree*). Using the metaphor of a library file card system for information exchanged in a discourse, Reinhart suggests that the topic—what the sentence is about—can be thought of as an entry in the system or the header of a file card under which information is filed. This information is the proposition expressed by a sentence, of which the focus is a part. Adopting this definition, we can say that in the subject focus conditions, the test sentence provides new information about the previously mentioned object, the elephant (namely who nudged him), so that the object is the topic. In the object focus conditions, it is the subject who is likely interpreted as the topic about which new information—the focus—is added. Finally, note that while all grammatical subjects were simultaneously agents and objects simultaneously patients, the inclusion of object clefts allowed us to partially compare the effects of subject and first mention preference in English: clefting fronts the object, which is otherwise always the second mention in a transitive sentence.

**TABLE 1:** Example illustrating experimental conditions.

Condition	Felicitous context question (Speaker B)	Test sentence (Speaker A response)
*Broad focus*	Yeah, I heard something happened. Do you know what?	The giraffe nudged the elephant beside the tree. He wanted to be friendly.
*Subject focus: cleft absent*	Yeah, I heard someone nudged the elephant beside the tree. Do you know who?	The GIRAFFE nudged the elephant beside the tree. He wanted to be friendly.
*Subject focus: cleft present*		It was the GIRAFFE that nudged the elephant beside the tree. He wanted to be friendly.
*Object focus: cleft absent*	Yeah, I heard the giraffe nudged someone beside the tree. Do you know who?	The giraffe nudged the ELEPHANT beside the tree. He wanted to be friendly.
*Object focus: cleft present*		It was the ELEPHANT that the giraffe nudged beside the tree. He wanted to be friendly.

*1. CAPITALS indicate prosodic focus marking. 2. Verb list: splashed, kicked, slapped, bit, hugged, bumped, scratched, squeezed, pinched, poked, kissed, pushed, slapped, tickled, hit, nudged, pet, shoved, cuddled, touched.*

Our primary goal was to investigate whether linguistic focussing influences ambiguous pronoun resolution. For example, do focussing cues help establish a subject and first mention preference, and do they hinder or override such preferences in the case of object focus? On the one hand, adults may have developed such robust preferences for the other inherently present cues, such as grammatical role, that linguistic focus appears to have no effect (such that focus cues are somewhat less activated, or dismissed as relatively less relevant and less reliable). On the other hand, if the linguistic focus cues are stable and used by adults, they should be activated and explain unique variance in their preferences by enhancing baseline subject looks within the subject focus conditions and hindering them in object focus conditions. Based on recent findings on the stability of prosodic focus marking irrespective of the use of clefts within general adult comprehension (Arnhold, 2021), we also explored whether adults use prosodic focussing cues regardless of cleft presence.

For children, many have suggested a reduced ability and experience in using multiple cues, for example, in their activation and suppression (Arnold et al., 2007; Theakston, 2012; Järvikivi et al., 2014; Allen et al., 2015; Hartshorne et al., 2015). If their general preference for the subject (and first mention) is weaker than adults’, as is suggested by the literature (Hartshorne et al., 2015), these preferences may be more likely to be modulated by focus cues relative to adults (Järvikivi et al., 2014). That said, children must have enough language experience with the focus cues and also have acquired the processing skills necessary to activate and use them accordingly. To assess this latter point, we incorporated independent measures of children’s working memory and language ability. Starting from a simple framework, we adopt a suggestion regarding referential forms in general (Niewland and Van Berkum, 2006; Serratrice and De Cat, 2018; Arnold et al., 2019; Langlois and Arnold, 2020; Qi et al., 2020), that strong memory and language skills should predict an ability to activate the most plausible referent (i.e., the interpretive preference displayed by adults). Therefore, if linguistic focus marking cues indeed modulate children’s subject and first mention preferences, any potential requirements to activate these cues and/or suppress less reliable cues should be more straightforward for high scorers in our working memory and vocabulary depth tasks.

## Materials and Methods

### Participants

Fifty-eight undergraduates from the University of Alberta completed the experiment, all of whom spoke English as their first language. Thirty-seven children (mean age 4;4; range = 3;1–6;3, 20 boys) completed the experiment and were recruited from preschools and daycares in the Edmonton, Alberta, region of Canada. Two children were excluded because they did not complete the eye-tracking portion of the experiment and one additional child was excluded due to equipment failure resulting in the loss of data. All children were monolingual English speakers with no reported language disabilities.

### Materials and Procedure

During the first session, all participants completed a pronoun interpretation visual world paradigm experiment, which lasted no longer than 20 min for adults and 30 min for children. During a second session, children’s memory and vocabulary depth were independently assessed, which took approximately 20 min in total. All assessments took place in a quiet setting within a university laboratory (adults) or preschool classroom (children). A trained research assistant led each session.

#### Pronoun Processing: Visual World Paradigm

**FIGURE 1:**
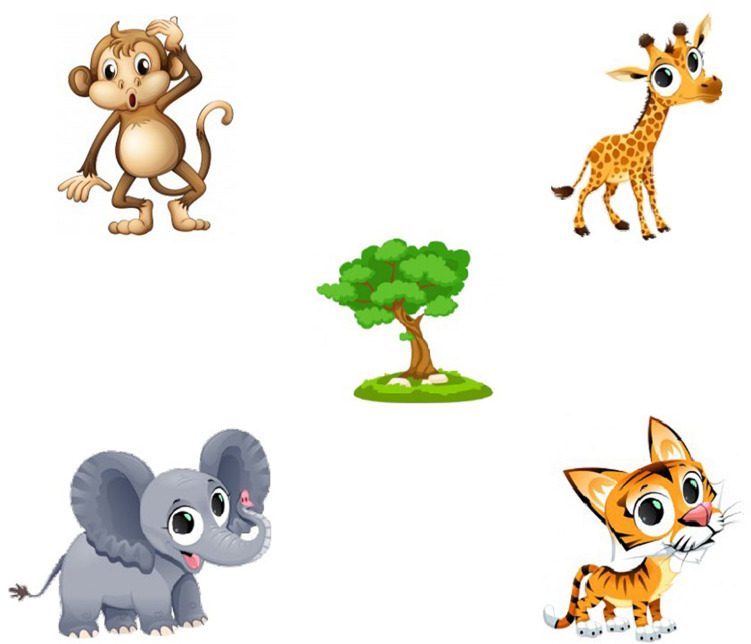
Example of experimental item screen presentation.

Twenty experimental items were constructed so that each item was a 3-turn dialogue between two speakers (one male and one female). There were five versions of each item in order to capture the five different experimental conditions: *subject focus-cleft absent, subject focus-cleft present, object focus-cleft absent, object focus-cleft present*, and lastly a *broad focus* condition where neither the subject nor object was focussed. Each item referenced a location and four animal characters, two of which were the critical subject or object and another two which served as distractors. The spoken dialogues were simultaneously presented with displayed images of all four animal characters, as well as the location which was included so that participants would fixate on it prior to the critical test region [see example display in Figure 1 and dialogue in (4)]. During the first turn, Speaker A introduced the four animal characters along with the location. In the second turn, Speaker B asked a question that provided a felicitous context to Speaker A’s upcoming answer. This question depended on whether the experimental condition focussed the subject, object, or neither (broad focus). In the third turn, Speaker A answered the question in line with one of the five experimental conditions, followed by a sentence starting with the critical ambiguous pronoun, *he*. Examples of all conditions are given in Table 1.

(4) Speaker A: *Yesterday at the zoo I saw a monkey, a giraffe, a tiger and an elephant.*
Speaker B: *Yeah I heard someone nudged the elephant by the tree. Do you know who?*
(subject focus conditions)
Speaker A: *It was the GIRAFFE that nudged the elephant by the tree. He wanted to be friendly.* (*subject focus-cleft present* condition).

Forty animals and 20 locations were used to create the different experimental items, with all corresponding images being selected from an internet source Freepik (2017). A unique transitive verb was also used for each item. Items were counterbalanced into five separate lists so that participants received only one version of each item.

The display screens counterbalanced the presentation of the four characters into one of the four screen corners, with the location always in the middle. Narrations were recorded in a natural manner, but with clear and consistent prosodic focus marking, by one male and one female Canadian speaker. SR Research software (2020) was used to programme and pseudorandomise the experiment in Experiment Builder, and also to run the experiment using the remote mode of an Eyelink portable duo (children) or Eyelink 1000plus (adults). The sampling rate was 500 Hz, recording gaze locations every 2 ms.

Each participant took part individually. Children first completed an animal familiarisation task, where they were asked to preview and name the dialogue characters one by one on the computer screen. All visual world sessions began with a calibration and validation procedure, which both required participants to fixate on five markers (a smiling *Mr. Sun* character) that separately appeared on the screen. If spatial accuracy errors for each marker exceeded more than 2 degrees, these procedures were repeated. This was followed with practice items in *broad focus* condition in order to ensure that participants understood the procedure. Participants listened to 20 dialogue sequences (from one of the five lists) while we recorded their eye movements toward characters on the screen. After each dialogue, the visual array of the characters and landscape remained on the screen, and the participant was asked to determine who they thought the ambiguous pronoun referred to (e.g., *Who wanted to be friendly*?). A blank white screen was then shown, and the next item only began once the participant fixated on the *Mr. Sun* marker positioned in the center of the screen (a drift-correct calibration check). If the drift-checking procedure found an error that exceeded 2 degrees of visual angle, the calibration procedure was repeated. The research assistant coded each offline response to a question by pressing a keyboard button that corresponded to the chosen character.

##### Design summary

A *broad focus* condition served as a baseline comparison for four experimental conditions that used non-contrastive narrow focus questions and prosodic focus marking on the subject or object. The prosodically focussed character was either further marked with an it-cleft, or was not (Table 1). The response variable was subject preference looks, which was calculated by subtracting looks to the object character from looks to the subject character. This was measured for a period of −200 to 2,200 ms from the onset of the ambiguous pronoun. However, it should be noted that, as is usually the case with English studies, a subject preference can be co-attributed to the agent being the subject argument in all our conditions. Agentivity was not part of the experimental design of the present study, so (high transitive agent-patient) verbs were counterbalanced across all five conditions, and could not influence the differences between conditions. Further, with the exception of the *object focus-cleft present* condition, subject preference can be co-attributed to its convergence onto first mention. In the subject focus conditions, the linguistic focus cues marked the subject, first mention, and agent. In the object focus conditions, the linguistic focus cues conflicted with other cues. This confliction was strongest in the *object focus-cleft absent* condition, where linguistic focus marked the object, second mention, and patient. In the *object focus-cleft present* condition, the object was fronted, so linguistic focus conflicted with grammatical role and semantic role cues (i.e., aligned to the object and patient).

#### Working Memory Assessment

To assess working memory, each child completed the Nebraska Barnyard task from an executive function battery by Wiebe et al. (2011). The task was administered on a touchpad screen and run in E-Prime 1.1 (Psychology Software Tools, 2018). This measure is a suitable assessment of memory for our age because the responses reliably display appropriate distributional properties from 36 months (Wiebe et al., 2011), and because 4-year-olds perform at floor on equivalently complex measures of working memory (Gathercole et al., 2004). Scoring was according to Wiebe et al. (2011).

#### Vocabulary Depth Assessment

Children’s vocabulary depth was assessed using an experimenter-adapted version of a word description task (see Hughes et al., 2005; Blewitt et al., 2009; Hadley et al., 2016). In this task, the child has to describe the meaning of concrete and abstract nouns, verbs, and adjectives spoken aloud by the researcher. Twenty words were selected based on age of acquisition data reported by Morrison et al. (1992). That is, we selected the words so that they were appropriate for our youngest age group, specifically 10 words acquired before and after 3 years of age. Two words were practiced - one by the experimenter and one by the child. Each word was introduced by the question *What is a(n) X?* Since the aim was to collect rich definitions, the experimenter asked a follow up question, for example *Can you show me or tell me anything else about X*, or *Do you know anything else about X?* The experimenter moved onto the next word when the child had provided their full answer, or if a child gave an incorrect response. Testing was discontinued when four incorrect responses were made in a row. Responses were transcribed by a research assistant present during the session, and were also audio recorded for cases of uncertainty. The task aims to tap into semantic and contextual knowledge, rather than metalinguistic knowledge of the word like structure or form (Snow et al., 1991). This also enabled a rich scoring scheme.

##### Coding and scoring

The coding scheme was developed by Hadley et al. (2016; an adapted version of Hughes et al., 2005; Blewitt et al., 2009). Responses were coded in terms of information units that met any of eight categories. These were perceptual properties (e.g., *a pond is blue*); functional properties (e.g., *a stool is so you can stand on it and wash your hands*); parts (e.g., *a pond has little waves*); superordinate category (e.g., *a weed is a big spiky plant*); synonyms (e.g., *an accident is done by mistake*); gestures (e.g., *fetching: child makes a motion similar to throwing a stick*); meaningful context/factual (e.g., *a shield is something you hold in your hand*); basic context (e.g., *pond: the duckling is with its mommy duck*). Each information unit was then scored as one point apart from basic context (0.5). To avoid item-driven total scores, scoring for a single item was capped at two points (i.e., the maximum total raw score was 40). Three research assistants coded all the responses separately. The intra-class correlation coefficient was computed to assess the agreement between three raters. Under criteria set by Koo and Li (2016), the consistency between the three raters was rated excellent, using two-way random effect models, Intraclass Correlation Coefficient = 0.97, *p* < 0.01.

## Results

### Results: Offline Data

#### Treatment

Coding identified whether the response corresponded to the subject character (“1”) or the object character (“0”), and a missing “NA” was coded if a distractor was selected. Distractors were selected 6 times by adults (<1% of responses) and 91 times by children (13% of responses).

#### Analysis

Generalised Linear Mixed-effects Models (GLMMs) (Baayen et al., 2008; Barr et al., 2013) were fitted to the data in the R statistics environment (R Core Team, 2019) using the lme4 package (Bates et al., 2014). Condition was entered as a fixed effect with broad focus set as the reference level, and the likelihood ratio test (Pinheiro and Bates, 2000; Barr et al., 2013) was used to test whether the random effects were warranted by superior model fit to data. Random effects for the final adult model included random intercepts of the subject and item; whereas the final child model only required random intercepts for subjects.

Descriptive statistics indicated a ceiling subject preference by adults in all conditions other than *object focus-cleft present* [Means (± SD): broad = 0.9 (0.48), *subject focus-cleft-present* = 0.91 (0.28), *subject focus-cleft absent* = 0.9 (0.31), o*bject focus-cleft-present* = 0.65 (0.48), *object focus-cleft-absent* = 0.85 (0.36)]; and a tendency to prefer the subject by children in all conditions other than o*bject focus-cleft-present* [broad focus = 0.66 (0.48), *subject focus-cleft present* = 0.73(0.44), *subject focus-cleft absent* = 0.75 (0.43), o*bject focus-cleft present* = 0.50 (0.50), *object focus-cleft absent* = 0.66 (0.40)]. For both groups, o*bject focus-cleft present* had a significant effect such that subject preference was reduced: adults: *b* = −1.97 (SE = 0.29), *t* = −6.68, CI −2.54 to −1.39; children: *b* = −0.84 (SE = 0.29), *t* = −2.95, CI −1.40 to −10.28. The other conditions were not significantly different to *broad focus*, as indicated by their *t*-values not exceeding 2, and confidence intervals not passing zero (Baayen, 2008). The Supplementary Material Section 1 provides the full inferential statistics of the final model for adults and children.

### Results: Online Data

#### Treatment

**FIGURE 2:**
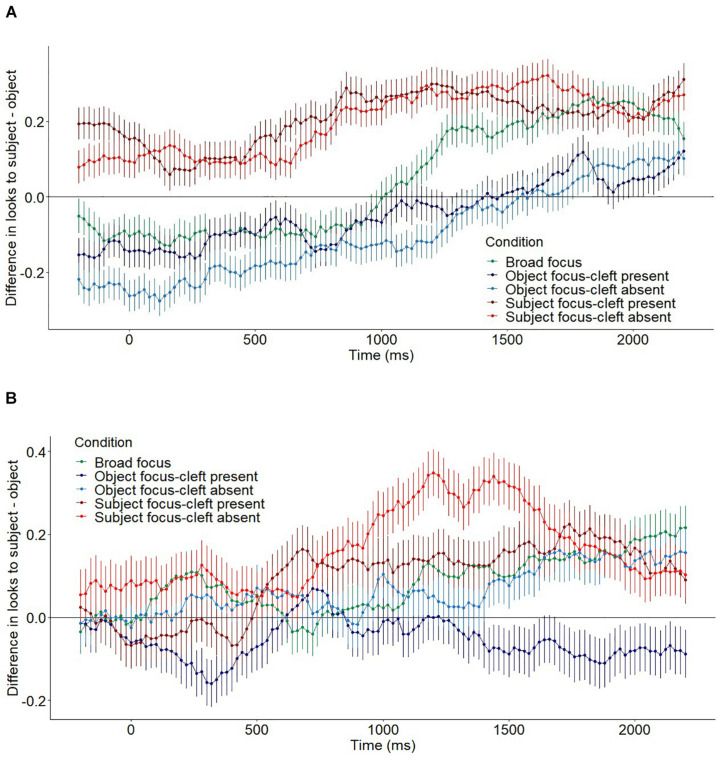
Fixation probabilities to the subject (the object subtracted from fixations to the subject, per condition [by time in ms; 0 ms = pronoun onset]). Top: adults **(A)**; bottom: children **(B)**. A positive score indicates subject preference whereas a negative score indicates object preference. Error bars indicate standard errors.

The raw gaze data was pre-processed in the VWPre package (Porretta et al., 2018). The time course window was set to 200 ms prior to the onset of the ambiguous pronoun, followed by a critical region of 2,200 ms. Note that it takes around ∼200 ms for the pronoun to be completed and an additional ∼200 ms to plan an eye movement (Matin et al., 1993), so any effects occurring before 400 ms should not be attributed as a direct effect of hearing the pronoun (see section “Discussion”). Figure 2 presents the raw data for adults (Figure 2A) and children (Figure 2B), with subject preference looks as the response variable (calculated by subtracting the proportion looks to the object character from the proportion looks to the subject character). A subject preference value of 0 indicates equal looks to the subject and object entities, while positive values indicate a preference for the subject and negative values a preference for the object.

#### Fitting and Evaluation of Our Main Models

We report a series of Generalised Additive Mixed Models (GAMMs; see van Rij et al., 2019a) fitted separately to the data for children and adults, using the package mgcv (Wood, 2017) in R (R Core Team, 2019). GAMMs are a non-linear extension to mixed-effects regression methods (GLMMs; Baayen et al., 2008), and are particularly beneficial when analysing time series data, given that the response variable in time series data does not typically display a linear increase or decrease. Thus by using GAMMs, we can determine when exactly predictors have an effect on the response variable. In GAMMs, smooth functions (Wood, 2017) afford the non-linear modelling of predictor terms, allowing the regression line (or interaction surface) to “wiggle” if required by the data.

It is not recommended for GAMMs to analyze fixation proportions, so they were logit-transformed using the function transform_to_elogit, which distributes the values symmetrically around zero and provides an unbounded measure for the analysis (Barr, 2008). Thus, the response variable for our GAMM analysis was subject preference looks as empirical logits (i.e., e-logits), calculated by subtracting the e-logit looks to the object character from the e-logit looks to the subject character.

To account for variation in participants and items, GAMMs allow for random intercepts and slopes (just as in linear mixed effects modelling), as well as random factor smooths, which are unique to GAMMs and adjust the shape of the regression line or interaction surface with a potentially non-linear trend for each participant and item (naturally incorporating random intercepts and slopes). We followed recommendations of van Rij et al. (2019b) to fit each model with by-participant and by-item random smooths to the effects of time, as well as by-event (each unique participant-item response) intercepts and slopes to time. Accounting for more error variance generally reduces the residual errors, resulting in a better model fit to the data. Our complex random structure is also one solution to autocorrelation, which was further accounted for by using an AR1 model (see Wood, 2017).

We followed the recommendations of the majority of GAMM literature for reaching our reported best-fit models by using a backward-fitting stepwise elimination procedure (e.g., van Rij et al., 2019a). That is, all terms were included in an initial model and then the contribution of each term was evaluated using three criteria deemed to complement each other: (i) the estimated *p*-value (based on the *F* statistic) in the model summary; (ii) the Maximum Likelihood (ML) score comparison of model variants using the compareML function in the itsadug package (van Rij et al., 2020); and (iii) visual inspections of the model, again using functions from the itsadug package.

Experimental condition was fitted to the response variable as a five-level categorical predictor (akin to a linear fixed effect term): the levels included *broad focus* as the reference, *subject focus-cleft present*, *subject focus-cleft absent*, *object focus-cleft present*, and *object focus-cleft absent* sentences. An interaction between condition and the (continuous) time course was also fitted as a non-linear smooth.

#### Adult Main Model: Summary Statistics and Visualisations

**FIGURE 3:**
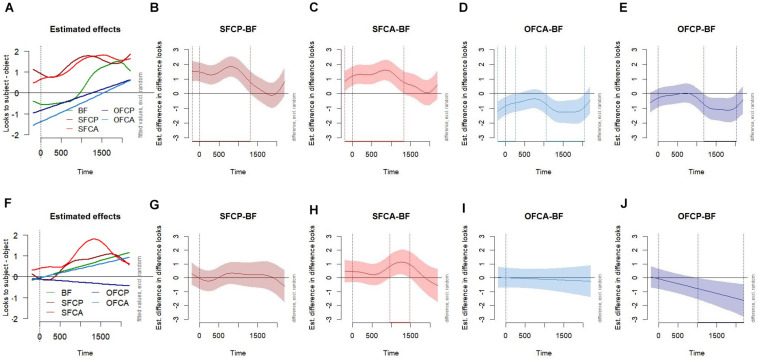
Visualisation of the summed effects derived from the “Main” models of adult **(A–E)** and 3- to 6-year old **(F–J)** fixation patterns, with the random effects set to zero. **(A,F)** Smooth terms for each time by condition term. **(B–E,G–J)** Difference plots visualising the difference between the *broad focus* condition with each other condition. The difference plots are coloured consistent with the grand means (Figure 2) and smooth terms plot **(A,F)**, and have been abbreviated from left to right “SFCP” = *subject focus-cleft present* (dark red), “SFCA” = *subject focus-cleft absent* (red), “OFCA” = *object focus-cleft absent*, (blue), and OFCP = o*bject focus-cleft present* (navy blue).

Inferential statistics for the main model of the adult data are provided in Table 2. The first rows provide parameter coefficients that can be interpreted in a similar fashion to GLMMs, such that, relative to the *broad focus* reference level, a positive *Estimate* value indicates a stronger subject preference and a negative estimate value indicates a weakened subject preference. These values only tell us about the difference between conditions when Time is equal to 0 (pronoun onset), so we move to the smooth terms for interpretation. The edf (effective degrees of freedom) column indicates the “wiggliness” of the regression line, where a value greater than 1 indicates non-linearity. The smooth term *p* values indicate whether the regression line significantly differs from 0 at any point in the time course (where 0 indicates equal looks to the subject and object). In order to interpret the shape of the regression lines and determine when they differ from 0, plotting is necessary. The summed effects for all conditions are visualised in Figure 3A (see Supplementary Material Section 2, for more detailed figures).

**TABLE 2:** Summary statistics of the “Main” Generalised Additive Mixed Model for adults.

Parametric coefficients	Estimate	Std. Error	*t*	Pr(>|*t*|)
(Intercept)	0.35	0.25	1.40	0.16
Object focus-cleft present	−0.52	0.31	−1.66	0.10
**Object focus-cleft absent**	**−0.83**	**0.31**	**−2.70**	**0.01**
**Subject focus-cleft present**	**1.01**	**0.31**	**3.26**	**<0.01**
**Subject focus-cleft absent**	**0.95**	**0.31**	**3.05**	**<0.01**
**Smooth terms**				
	**edf**	**Ref.df**	**F**	***p*-value**
**s(Time):Broad focus**	**6.36**	**7.65**	**6.48**	**<0.01**
**s(Time):Object focus-cleft present**	**1.01**	**1.02**	**1.97**	**<0.01**
**s(Time):Object focus-cleft absent**	**1.09**	**1.17**	**17.24**	**<0.01**
**s(Time):Subject focus-cleft present**	**5.92**	**7.24**	**3.45**	**<0.01**
**s(Time):Subject focus-cleft absent**	**5.21**	**6.51**	**2.81**	**0.01**
*Random effects*				
s(Time,Subject)	253.56	521.00	2.13	<0.01
s(Time,Item)	75.93	179.00	2.61	0.01
s(Event)	734.48	1095.00	6.75	<0.01
s(Time,Event)	628.62	1095.00	5.23	<0.01

*Reporting parametric coefficients of sentence condition; and the smooth terms of sentence condition by time, with by-Subject and by-Item random smooths to time, and by-event random intercepts and slopes to time. R-sq.(adj) = 0.49; Deviance explained = 49%; –ML = 180,590; n = 133,100. Values in bold indicate that the predictor is significant at p < 0.05.*

Across all conditions, a subject preference generally increased across the time course. In the *broad focus* condition, an early object preference occurred from −200 to 750 ms; whereas a subject preference occurred at 1,100–2,200 ms. For both subject focus conditions, a subject preference was significantly different from zero from −200 to 2,200 ms. Since we should not expect to see evidence of pronoun influence until at about 400 ms, this suggests attentional preferences prior to the pronoun. However, the slope increases from around 500 ms, suggesting that pronoun effects are likely occurring in combination with such attentional effects (see Discussion). This interpretation also applies to the object focus conditions, where an immediate object preference was present from −200 ms but also extended to the region we would expect for pronoun effects: −200 to 860 ms in the *object focus-cleft present* condition, and −200 to 1,500 ms in the *object focus-cleft absent* condition.

Figures 3B–E display the difference plots, which are essential for examining whether smooths of each experimental condition significantly differ from the *broad focus* baseline. Here, the subject preference value for each comparison condition (i.e., subject minus object looks) is subtracted by the corresponding value for the *broad focus* condition. In each panel, the solid line represents the estimated difference between the comparison conditions (with shading for pointwise 95% confidence intervals), and the dashed vertical lines highlight any time window(s) for which this difference is significant. A positive value indicates that the subject preference was greater relative to the broad focus condition, as revealed in the significant windows of the *subject focus-cleft present* (−200 to 1,300 ms) and *subject focus-cleft-absent* (−200 to 1,320 ms) plots, which have effects of a similar magnitude and time course. A negative value indicates that the subject preference was reduced relative to the *broad focus* condition, as in the significant windows of the *object focus-cleft absent* (−200 to 260 ms and 1,060 to 2,050 ms) and o*bject focus-cleft present* (1,180–2,030 ms) plots, which again have a similar magnitude and time course to each other.

The Supplementary Material Section 3 provides subsidiary analyses that followed up on the similarity of linguistic focus effects with and without cleft presence (based on timing, magnitude, and their overall visual similarities in Figure 3A). Indeed, when the *subject focus-cleft absent* condition was used as the reference level, there were no significant differences between that condition and *subject focus-cleft present* condition; and correspondingly, there was no significant difference between object focus conditions when *object focus-cleft absent* was used as the reference level.

Supplementary Material Section 4 provides another subsidiary analysis (with full interpretation) that was run with the *object focus-cleft present* condition as the reference level, and first mention preference looks as the response variable (calculated by subtracting looks to the second mentioned character from looks to the first mentioned character). The motivation for this analysis is that the *object focus-cleft present* condition is an exception to the other conditions, such that the object is fronted as the first mentioned entity (in all other conditions the object is second mentioned). Therefore this analysis enabled a check that the aforementioned effects were a combination of focus with grammatical role *and* order of mention, rather than only one of these factors: If the *object focus-cleft present* sentences do not differ in first mention preference relative to other conditions, it would indicate that the aforementioned subject preferences were driven by the combination of focus with order of mention, and not in combination with grammatical role. However, the results support the presence of a subject preference in addition to a first mention preference. That is, online looking preferences are stronger the more that focus, subjecthood and first mention cues are aligned.

#### Models for Children: Summary Statistics and Visualisations

##### Main model

Using the same backward fitting procedure as outlined for the adult main model, a series of GAMMs were built for the child data to reach the main model. Inferential statistics for the main model are provided in Table 3. We turn directly to the visualisations of these smooth terms in Figure 3F (see Supplementary Material Section 5, for by-condition plots), representing the interaction between condition and (continuous) time course. A significant subject preference was present in the *broad focus* condition starting from much earlier than for adults, from 600 to 2,200 ms, and also for a similar time window in the case of the *subject focus-cleft present* sentences (540–2,200 ms). In the *subject focus-cleft absent* condition there was a significant subject preference present even prior to the onset, which lasted for the entire duration (−100 to 2,200 ms). Crucially, starting around 600 ms there was a steep increase in the slope, which likely reflects an effect of the pronoun that we see in the previously mentioned conditions. Conversely, the two object focus conditions did not significantly differ from zero. The time by condition difference plots in Figures 3G–J revealed that, relative to the *broad focus* sentence, there was a significantly greater subject preference on hearing *subject focus-cleft absent* sentences (964–1,473 ms); and that the subject preference was significantly reduced in *object focus-cleft present* sentences (1,012–2,200 ms). *Subject focus-cleft present* and *object focus-cleft absent* sentences did not differ from *broad focus*.

**TABLE 3:** Summary statistics of the “Main” Generalised Additive Mixed Model for 3- to 6-year-olds.

Parametric coefficients	Estimate	Std. Error	*t*	Pr(>|*t*|)
(Intercept)	0.51	0.29	1.76	0.08
**Object focus-cleft present**	**−0.77**	**0.39**	**−1.99**	**0.05**
Object focus-cleft absent	−0.10	0.39	−0.26	0.79
Subject focus-cleft present	0.08	0.39	0.22	0.83
Subject focus-cleft absent	0.47	0.39	1.20	0.23
**Smooth terms**				
	**edf**	**Ref.df**	**F**	***p*-value**
**s(Time):Broad focus**	**1.02**	**1.04**	**4.65**	**0.03**
s(Time):Object focus-cleft present	1.03	1.05	0.28	0.59
s(Time):Object focus-cleft absent	1.01	1.02	2.89	0.09
**s(Time):Subject focus-cleft present**	**5.36**	**6.70**	**2.87**	**0.01**
**s(Time):Subject focus-cleft absent**	**5.67**	**7.02**	**4.57**	**<0.01**
*Random effects*				
s(Time,Subject)	155.30	323.00	2.97	<0.01
s(Time,Item)	84.50	179.00	3.12	<0.01
s(Event)	386.01	573.00	7.86	<0.01
s(Time,Event)	352.00	573.00	6.88	<0.01

*Reporting parametric coefficients of sentence condition; and the smooth terms of sentence condition by time, with by-Subject and by-Item random smooths to Time, and by-event random intercepts and slopes to Time. R-sq.(adj) = 0.48; Deviance explained = 49%; –ML = 83,749; n = 69,938. Values in bold indicate that the predictor is significant at p < 0.05.*

A series of subsidiary child models were run as a follow up to the main model. Supplementary Material Section 6 provides summary statistics and respective difference plots for models investigating whether there was a difference between cleft present versus cleft absent conditions within each focus location. When the *subject focus-cleft absent* condition was set as a reference, it did not significantly differ from the *subject focus-cleft present* condition. However, that absence of a significant statistical difference within subject focus conditions should be interpreted with caution—the main model reported that subject focus marking enhances subject preference (relative to the *broad focus* baseline) when operationalised as prosody alone, but not when in combination with a cleft. Another subsidiary model employed the *object focus-cleft absent* condition as the reference condition and confirmed that *object focus-cleft present* sentences were significantly more likely to be associated with a reduced subject preference, resulting in more looks to the object than *object focus-cleft absent* (1351–2,200 ms).

##### Response variable set to first mention preference

The main model finding that, unlike adults, *object focus-cleft absent* (object = second mention) sentences did not significantly reduce children’s subject preference relative to the *broad focus* condition, suggests that the significant difference between *broad focus* and *object focus-cleft present* (object = first mention) sentences was more driven by a first mention preference than focussing of the object *per se*. This was confirmed in a subsidiary analysis that was run with first mention preference looks as the response variable (Supplementary Material Section 7). As with the equivalent adult subsidiary model (see end of section “Fitting and Evaluation of Our Main Models”), the other difference plots supported that preferences are strongest when focus is fully aligned onto the subject *and* first mention.

##### Models for children incorporating individual difference measures

For the children’s data, we ran exploratory models which additionally incorporated an individual difference predictor (either age, memory, or vocabulary depth; each scaled and centred). Individual difference predictors were run in separate models, as is recommended for collinear predictors in GAMMs (van Rij et al., 2019a): all correlations were significant (*p* < 0.05) and medium to strong in size (memory and vocabulary: *r* = 0.51; memory and age: *r* = 0.44; age and vocabulary: *r* = 76). The previously reported backward-step elimination procedure was used to determine the best-fitting models. Crucially, the best-fitting GAMM for both the memory model and the vocabulary model included a two-way interaction between the respective individual difference measure and the focus condition, as well as a three-way interaction between the time course, the respective individual difference measure and the focus condition (discussed in further detail below). We do not report the best-fit age model because age terms were not sufficient in meeting model inclusion criteria via the model summary terms and visualisation. Below, we only interpret the terms involving individual differences, as our previous “main” model already examined the time by condition terms under the same random effects structure.

###### Memory model

**FIGURE 4:**
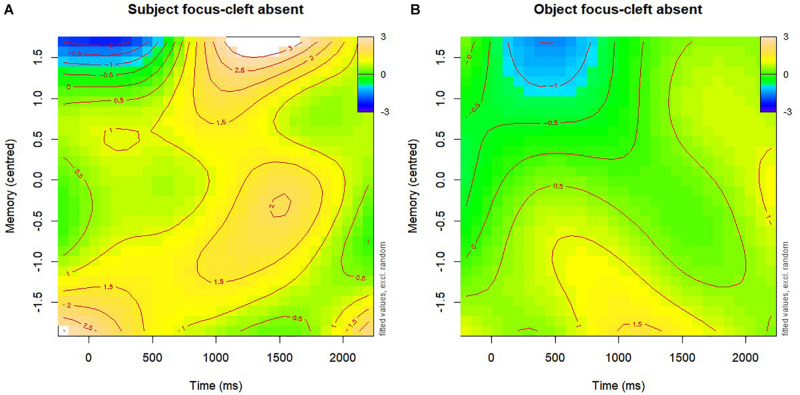
Contour plots of three-way interactions between Time (x-axis), Memory Scores on the Nebraska barnyard task (y-axis) and subject focus-cleft absent **(A)** or object focus-cleft absent **(B)** conditions. Yellow indicates a subject preference, whereas green indicates a reduced subject preference. Blue colours represent object preference, but these are too minor to interpret.

Table 4 reports the summary statistics of the best-fit memory model. There was a significant memory ability modulation of time course effects in the *subject focus-cleft absent* condition, as visualised in the Figure 4A contour plot. Contour plots are read like topographic maps, and a description of how to interpret our formatting is provided within the Figure caption. For *subject focus-cleft absent* sentences, children who had very low memory scores displayed a strong subject preference limited to within the first 500 ms, whereas children with middle and high memory scores showed an increased subject preference from around 1,000 ms onward. It was unexpected that children with poorer memory ability would display an enhancement of subject preference for these sentences at an earlier point in time course (prior to 500 ms); however, since pronoun-related effects more typically occur in adults from around 500 ms onward, we return to this point in the discussion as an effect that is more likely explained by attentional-only effects that occur prior to the pronoun. Importantly, the later time window for children with middle to high memory scores corresponds to our earlier report that the *subject focus-cleft absent* condition displayed a significant enhancement of subject preference relative to *broad focus* sentences from 964 to 1,473 ms. Summary statistics also indicated a significant memory modulation of the time course effects of *object focus-cleft absent* sentences. This is illustrated by Figure 4B, where children with low memory scores displayed a tendency to prefer the subject after around 700 ms, whilst children with medium and high memory scores only displayed tempered subject preferences. Supplementary Material Section 8 shows that both these three way interactions held, and that no additional effects appeared, when the response variable was first mention preference looks instead.

**TABLE 4:** Summary statistics of the “Memory” Generalised additive mixed model for 3- to 6-year-olds.

Parametric coefficients	Estimate	SE	*t*	*P*
(Intercept)	0.53	0.30	1.74	0.08
**Object focus-cleft present**	**−1.12**	**0.42**	**−2.68**	**0.01**
Object focus-cleft absent	−0.22	0.41	−0.53	0.60
Subject focus-cleft present	0.06	0.41	0.14	0.89
Subject focus-cleft absent	0.53	0.41	1.29	0.20
**Smooth terms**				
	**edf**	**Ref.df**	**F**	***P***
s(Time):Broad focus	1.01	1.01	2.00	0.16
s(Time):Object focus-cleft present	1.03	1.06	0.24	0.62
s(Time):Object focus-cleft absent	1.01	1.02	2.46	0.11
s(Time):Subject focus-cleft present	3.42	4.40	1.72	0.12
**s(Time):Subject focus-cleft absent**	**4.75**	**6.03**	**2.86**	**0.01**
s(Memory):Broad focus	1.00	1.01	0.07	0.79
s(Memory):Object focus-cleft present	1.00	1.00	0.23	0.63
s(Memory):Object focus-cleft absent	1.01	1.01	1.32	0.24
s(Memory):Subject focus-cleft present	1.00	1.00	0.10	0.75
s(Memory):Subject focus-cleft absent	1.00	1.00	0.11	0.74
ti(Time, Memory):Broad focus	11.40	13.56	0.93	0.44
ti(Time, Memory):Object focus-cleft present	6.14	8.09	1.22	0.27
**ti(Time, Memory):Object focus-cleft absent**	**8.00**	**1.10**	**2.21**	**0.01**
ti(Time, Memory):Subject focus-cleft present	7.34	9.51	0.84	0.57
**ti(Time, Memory):Subject focus-cleft absent**	**11.90**	**13.94**	**3.48**	**<0.01**
*Random effects*	11.40	13.56	0.93	0.44
s(Time,Subject)	12.16	268.00	2.24	<0.01
s(Time,Item)	75.69	179.00	2.47	<0.01
s(Event)	319.05	476.00	7.87	<0.01
s(Time,Event)	289.88	476.00	6.87	<0.01

*Reporting parametric coefficients of sentence condition; and the smooth terms of each sentence condition by time, each sentence condition by Memory, and a three-way interaction. Random effects of Time were by-Subject and by-Item random smooths, and by-event random intercepts and slopes. R-sq.(adj) = 0.49; Deviance explained = 50%; –ML = 69,390; n = 58,806. Values in bold indicate that the predictor is significant at p < 0.05.*

###### Vocabulary model

Summary statistics and visual plotting revealed that vocabulary modulated the interaction between time and *subject focus-cleft absent*, displaying a very similar pattern to that modulated by memory (see Supplementary Material Section 9). However, vocabulary depth did not significantly modulate time course effects on *object focus-cleft absent* sentences. When the response variable was first mention preference looks, vocabulary depth held its significant modulation of *subject focus-cleft present* sentences, and also then significantly modulated the time course effects of *object focus-cleft present* sentences (as in memory interactions). The discussion further explores the similarities between our individual measures.

## Discussion

In a visual world eye tracking study, we investigated whether adults and children use linguistic focussing devices, embedded within a felicitous discourse, to help resolve ambiguous personal subject pronouns. Linguistic focus on the grammatical subject or object of the sentence prior to the pronoun was operationalised as prosodic marking either in the presence or absence of it-clefts. This linguistic focus served a pragmatic purpose to focus a referent from prior discourse that involved a non-contrastive question (e.g., *Yeah I heard someone nudged the elephant by the tree. Do you know who?*). Our data revealed that linguistic focussing via prosodic marking, embedded within a felicitous discourse, impacted both adult and child real time pronoun processing. The presence of it-clefts had no significant effect on adult online processing once prosodic focus was taken into account; and this partially extended to child online preferences such that subject it-clefts had no significant influence. Object focussed it-cleft sentences significantly reduced an online baseline subject preference by children, which we attribute to the object moving to the first position in the sentence. The condition with object it-clefts was also the only one to affect the offline measure of pronoun interpretation in both adults and children, significantly reducing the subject preference. Taken together with our additional finding that children’s interpretive preferences were modulated by individual differences in memory and language ability, our observations are informative to how adults and children use multiple cues to appropriately represent entities within their discourse models of the text meaning (Zwaan and Radvansky, 1998).

The influence of discourse context and linguistic focus via prosodic marking on online interpretive preferences was over and above well-established cues like subjecthood, first mention and agentivity, and this was particularly robust for adults. First, conditions that prosodically marked the subject as focussed significantly enhanced subject preference looks relative to the *broad focus* condition. Second, prosodic marking of the object significantly reduced the subject preference relative to the *broad focus* condition. Significant focussing effects for adults were found regardless of whether clefts were present. This finding may appear to contradict some previous studies that reported an effect of clefts on pronoun resolution (Cowles et al., 2007; Foraker and McElree, 2007; Colonna et al., 2015), but as those studies did not control for the effect of prosodic focus marking, it is possible that their cleft effects were due to concomitant prosodic focus marking (whether present in the spoken stimuli or mentally added by readers as silent prosody). Independently testing the effect of prosody, our results correspond with previous findings that clefts do not appear to influence adult pronoun processing *per se* (Kaiser, 2011; Colonna et al., 2012; Colonna and Hemforth, 2014; Järvikivi et al., 2014). It is also in line with general adult literature showing that prosodic marking is undiminished in the presence of clefts (Arnhold, 2021), that prosodic focus marking on its own provides a greater memory advantage than clefts on their own (Cowles et al., 2007; Kember et al., 2019), and that prosodic focus marking is ubiquitous, but the use of clefts to mark focus is relatively rare in spoken English (Sánchez-Alvarado, 2020). Importantly, clefts are not simply an alternative and equivalent to prosodic focus marking, but have long been recognised as complex constructions with a specific combination of syntactic, semantic and pragmatic characteristics (Declerck, 1988; Kiss, 1999; Hedberg, 2000; Drenhaus et al., 2011; DeVeaugh-Geiss et al., 2015; Destruel and Donaldson, 2017). Still, focus marking is a primary function of clefts, as well as of prosody (which usually seems to accompany clefts and help them perform this function, see Arnhold, 2021). In turn, the focused parts of utterances are boosted in terms of attention and memory (Cutler and Foss, 1977; Cutler and Fodor, 1979; Birch and Garnsey, 1995; Foraker and McElree, 2007; Kember et al., 2019; Káldi and Babarczy, 2021); in other words they become more activated and central in the listener’s mind. It is commonly assumed that pronouns are interpreted as referring to the entities that are most activated and central at a given moment (Gundel et al., 1993; Zwaan and Radvansky, 1998; Hartshorne et al., 2015). Therefore, it can be asked to what extent looking preferences detected in our data reflect (i) ongoing attention (or activation) processes that would affect looking preferences regardless of whether a pronoun was present; or (ii) pronoun-driven preferences triggered by the online processing and offline interpretation of the pronoun itself, which are also influenced by the preceding context but introduce constraints specific to the pronoun rather than being merely additive. Third, an important distinction is between the online processing of pronouns considering various cues and the final interpretation of pronouns as reflected in offline responses. We shall first discuss the differentiation between (i) and (ii) before considering this last point.

The time course for linguistic focus effects in adults provides a lens into understanding to what extent they can be interpreted as pronoun-driven. Specifically, it takes at least 400 ms to hear the pronoun and to plan an eye movement, so any significant effects occurring prior to that time cannot not be interpreted as a direct effect of hearing the pronoun. The main analysis of adults revealed that all four focus conditions significantly differed to chance from −200 ms; and this time course also applied to when conditions were compared to *broad focus*, with the exception of *object focus-cleft present* (beginning at 1,180 ms). Note that the subject preference in the *broad focus* condition differed from chance from 1,100 ms onward, corresponding to the timing in equivalent sentences used by Hartshorne et al. (2015; also see Song and Fisher, 2005: from 1,000 ms), confirming that this was the most representative baseline of adult interpretive preferences. The effects from −200 ms to (at least) 400 ms can be interpreted in various ways. One could posit these are attention-only effects, such that the linguistically focussed entity from the prior sentence was still being attended. This particularly could be proposed for the object focus conditions, which experienced their highest magnitude of looks (to the object) at −200 ms and then linearly reduced toward chance level looks. However, the significant effects for each focus condition extended well beyond 400 ms, so an attention-only explanation should be extended to incorporate pronoun-related effects. That is, attentional effects might occur on their own at an early time point (−200 ms to at least 400 ms), but some of those cues continue to be, or become more, relevant in processing the pronoun, for which the subject is always the target attachment (as indicated by offline results; also see Introduction). This is well exemplified by the subject focus conditions, which displayed a subject preference from the beginning of the analysis window, with an additional steep slope increase in subject preference beginning from around 400 ms onward. Foraker and McElree (2007; also see Káldi and Babarczy, 2021) showed that linguistic focus, specifically clefting, renders the referent in focus more available (facilitating its memory representation), but not more accessible (not affecting the speed of processing). That is, the pronoun is not merely matched to the content in focal attention. A metaphor for this is a target, which is easier to hit when it becomes larger, even though it is not moved any closer. According to this line of thinking, focussing increases the likelihood of establishing reference, thus resulting in the observed difference in the eye movement record in terms of the overall cumulative proportion of looks to (in our case) the subject. Therefore, focussing aids processing in the sense of establishing reference to the subject antecedent more easily. Crucially, this should not be interpreted as suggesting that focus changes the relative preferences during processing or the final interpretation of the pronoun. Rather, the influence of focus is that it makes the subject antecedent - which was always the target - more available. In line with this, as we have shown, we see no increase in the final offline preferences: focus does not change how people interpret the dialogues and the referential relations in them. It just makes establishing those relations that much more likely or easier. Whilst some details of the explanation necessarily remain open here, these findings provide strong evidence for linguistic focus having an effect on online processing of the pronoun itself, as well as for other individual and overlapping language interpretation processes like attention being reflected in looking preferences before and during pronoun processing.

Turning to the third question of how online looking preferences (reflecting pronoun processing) correspond to offline responses (reflecting final pronoun interpretation), it is noteworthy that only the *object focus-cleft present* condition differed from the baseline condition by showing a reduced offline subject preference. Focussing the subject did not have a significant effect on offline responses for adults or children, whether operationalised as prosodic marking or clefting. This suggests that even though listeners attend to focus during online processing, as evidenced by their looking preferences (for discussion of child data, see below), our data did not point to focus having a significant effect on the final pronoun interpretation. This contradicts the frequent suggestion that focussed words are more likely pronoun referents, which has been advanced based on two distinct, though partly overlapping concepts of focus (also see Gundel et al., 1993; Lambrecht, 1994, p. 201; Huang, 2000, p. 250; Goldberg, 2006, p. 136). First, based on what could be termed psychological or attentional focus, it has been suggested that focussing brings a referent to the centre of attention and thereby makes it a more accessible antecedent for a pronoun (Linde, 1979; Grosz and Sidner, 1986; Gundel et al., 1993). Second, literature using the linguistic or information structural meaning of focus has stated that the focus of one sentence often becomes the topic of the next sentence (van Valin and LaPolla, 1997, p. 224; Goldberg, 2006, p. 136), i.e., the entity about which the next sentence provides new information (Reinhart, 1981). As unstressed pronouns are a common way to realise topics (Prince, 1981; Givón, 1983; Lambrecht, 1994, p. 132), this makes the focus of the previous sentence a likely referent for the pronoun in the next sentence. Thus, focus may signal an upcoming topic shift, as remarked in the context of pronoun resolution by Colonna et al. (2015; also see Patterson et al., 2017). However, within the information structure literature it is also clear that since every sentence has a focus, the focus does not always become the topic of the next sentence per default. Rather, there is a general tendency for topic continuity, meaning that the topic of a sentence will be the same as the topic of a preceding sentence (Givón, 1983; Lambrecht, 1994, p. 132). Consistent with this, the present data do not suggest that focus is regularly interpreted as signalling a topic shift across sentence boundaries causing a preference to interpret the focused entity as the pronoun referent. Rather, our data suggest that while focussing may have the potential to signal an upcoming topic shift, listeners do not assume this as a default. In the absence of further evidence that the pronoun was referring to the focussed referent, our participants robustly interpreted the pronoun as referring to the first-mentioned entity of the previous sentence, which was the subject in most conditions. Interestingly, this tendency held for conditions where the first-mentioned subject was the topic (*broad focus*, *object focus-cleft absent*), resulting in topic continuity across sentences, as well as for conditions where it was not (*subject focus-cleft present* and *subject focus-cleft absent*). While in a prototypical English sentence the first-mentioned entity is also the subject, topic, agent and preferred referent for subject pronouns in a following sentence, our data suggests that in cases of misalignment between cues, first mention, subjecthood and agentivity cues to pronoun interpretation override the tendency toward assuming topic continuity.

Children’s use of focus marking in online processing was most clearly demonstrated by their enhanced subject preference in the *subject focus-cleft absent* condition. Like adults, the influence of (subject) focus cues on children came through prosody, and was not enhanced by additional clefting. This finding may seem to conflict with the findings of Järvikivi et al. (2014) who found an effect of subject it-clefting with German children. Again, it is possible that the reason it-clefts influenced online interpretive preferences in that particular study may actually be the lack of other discourse and pragmatic cues or the fact that prosody was not controlled. In the present study, not only did the presence of a subject it-cleft fail to enhance subject preference relative to prosody alone, but when the it-cleft was present, there was no difference compared to baseline subject preference (as indicated by the *broad focus* condition), though a subsidiary analysis showed that the (visibly smaller) subject preference in the *subject focus-cleft present* condition was not significantly different to the *subject focus-cleft absent* condition. The absence of a clear effect of subject clefts in children could be explained in various ways, including the relatively low frequency of clefts impacting their use as cues to pronoun referents (Arnold, 2008, Arnold et al., 2019), the fact that the first contented noun (subject) is not the very first constituent in the sentence, which could reduce the first mention advantage (Kaiser, 2011) or the complex syntactic and pragmatic characteristics of clefts delaying their acquisition (Paul, 1985; but see Aravind et al., 2016).

For *object focus-cleft present* sentences, children displayed a reduced subject preference relative to their processing of *broad focus*, showing increased looks toward the prosodically marked and clefted object (and now first mentioned) entity. We do not attribute focus marking (prosody and cleft) as the lead cause for this preference, for several reasons. First, children’s performance with that same combination of focus cues in the *subject focus-cleft present* condition suggests that they did not use the linguistic function of clefts in their pronoun processing (also see Ambridge et al., 2006; Theakston et al., 2014). Second, when the object was focussed via prosody alone (*object focus-cleft absent*) there was no significant difference relative to the baseline *broad focus* condition. At this point, these two findings suggest that children only used prosodic focus on the object as a reliable cue when it converged onto the first mention. The importance of first mention was further demonstrated in our follow up subsidiary analysis which operationalised the response variable as looks to the first mention minus the second mention. This revealed that prosodic focus on the object and first mention did not enhance first mention looks relative to the *broad focus* baseline, indicating that the main model effect of *object focus-cleft present* sentences is more driven by children’s strong first mention preferences.

Adult and child offline responses painted a somewhat similar picture as online processing with regards to *object focus-cleft present* sentences: these were the only sentences that adults did not respond to with a ceiling subject choice (65%), and were the only sentences that children responded to at chance-level (50%). Therefore, both our online and offline findings for *object focus-cleft present* sentences corroborate that subject preference is typically more robust when aligned with multiple cues and is particularly hindered when the subject entity does not align with first mention. This is in line with previous studies using flexible word order languages (Järvikivi et al., 2005; Kaiser and Trueswell, 2008; Schumacher et al., 2017), and shows that both of these cues contribute to pronoun resolution in English.

A misalignment of cues may also explain why, unlike adults, prosody did not display a clear effect on children in object focus conditions. In these conditions, prosody was misaligned against established cues (subjectivity, first mention, and agentivity) and, compared to adults, children’s interpretive preferences are weakened by misaligned cues to a greater extent (Blything et al., 2021). Related, whilst sensitivity to linguistic functions of prosodic focus marking starts to appear between 3 and 6 years of age, it is still developing during this age window (Moore et al., 1993; Wells et al., 2004; Arnhold et al., 2016). Therefore, prosody is simply not as stable of a cue for these children, so may be activated relatively less than for adults. Indeed, this fits previous work showing that young children do not initially use cues in the same way as adults (Pyykkönen et al., 2010; Järvikivi et al., 2014; Goodrich Smith and Hudson Kam, 2015; Blything et al., 2021). The overall extent to which individual children resembled adults is likely dependent on a number of factors, ranging from whether they have the required language experience and developed the same robust preferences for established cues (e.g., subjecthood), as well as the processing skills required to appropriately activate and suppress multiple cues (Järvikivi et al., 2014; Hartshorne et al., 2015).

The time course modulation of children’s looking preferences in linguistic focus conditions relative to the *broad focus* condition reported significant effects only from around 1,000 ms post pronoun onset, strongly suggesting that focussing cues were generally having some form of an effect on the online processing of the pronoun itself, rather than on attention-*alone*. Additional models that incorporated individual difference measures of children’s memory and language also provided some time course effects that can be attributed to the presence of the pronoun. From around 500 ms onward there was a trend for children with medium to high scores in both working memory and vocabulary depth to be more likely to use prosodic marking to enhance (*subject focus-cleft absent*) or override (*object focus-cleft absent*) subject preferences from the pronoun region. However, the overall data show that, as we discussed for adults, children also displayed early effects that cannot be attributed to the pronoun. First, the *subject focus-cleft absent* condition differed to zero from −200 to 2,200 ms, meaning that there were preferences before any pronoun effects would be expected (at least 400 ms onward). Second, the three way interactions showed that, up until around 400 ms, only children with low scores in memory and language displayed an enhancement of subject preference for *subject focus-cleft absent* sentences. Exactly what such effects entail for these children, might be informed by general child language research. Studies have reported that young or low memory span children are less able to make use of their understanding for visual information to inform their interpretation of language, for example syntactic ambiguities (Trueswell et al., 1999; Weighall and Altmann, 2010; Zhang and Knoeferle, 2012; also see Cooper-Cunningham et al., 2020). Therefore, these children may be more amenable to (non-linguistic) attentional influences and less able to make use of the linguistic focussing function of prosody.

Importantly, whilst the subject preference in the *subject focus-cleft absent* condition differed to zero from −200 ms, it steeply rose after 500 ms. Therefore as suggested for adults, attentive modulation likely contributed to the effects on the medium and high scorers that occurred from the online processing of the pronoun itself. Collectively, this presents a complex picture for the role of memory and language, as well as attentional versus pronoun effects. However, it does provide support for our relatively straightforward prediction based on general literature for referential expressions, that variation in memory and language skills would predict children’s ability to interpret the most plausible referent of the pronoun (Zwaan and Radvansky, 1998; Serratrice and De Cat, 2018; Arnold et al., 2019; Langlois and Arnold, 2020; Qi et al., 2020). Our adult data showed that prosodic focus marking is attended to during pronoun processing, and this is more likely for children who have more available processing resources (Kidd, 2013; Järvikivi et al., 2014; Hartshorne et al., 2015).

### Limitations and Suggestions for Future Research

Our findings add to a growing body of recent work in experimental pragmatics that has reported on the importance of including language and context representative of the real world (e.g., Kidd et al., 2007; de Ruiter et al., 2018, 2020). Of course, in any experimental design there is a difficult balance to maintain between controlled methodology and ecological validity. One could argue that it would have been beneficial to have included a cleft condition in the absence of prosodic marking to more directly assess a previous finding that clefts can enhance subject focus for adults (Colonna et al., 2015) and to test for an interaction between clefting and prosodic marking. However, such a condition is not informative to real world situations because it-clefts rarely occur without prosodic marking (Arnhold, 2021). Similarly, since we embedded focus marking in a felicitous discourse context, we cannot separate the effects of prosodic focus marking (or clefting) from the effect of the preceding question, which already signaled the focus of the upcoming sentence. Separating these factors would, however, have come with significant downsides without necessarily leading to a more informative design. On the one hand, if the felicitous preceding discourse context is omitted, focus marking does not fulfill the same function as it does in its real-world usage, so that its effects are difficult to interpret (cf. discussion in the Introduction). On the other hand, the absence of prosodic focus marking when focus is clearly indicated with a preceding question likewise is unnatural and potentially confusing if prosody is controlled, e.g., flat, in spoken stimuli. This unnaturalness can be avoided by using written stimuli, but this does not necessarily eliminate prosodic effects, since readers are known to mentally impose silent or “implicit” prosody (see overview in Breen, 2014). Finally, our naturalistic, yet clear manipulation of focus via preceding questions means that the number of mentions within the dialogue was not the same for all referents. In our four focus conditions (see Table 1), the non-contrastive question explicitly named only the unfocused character of the test sentence (i.e., the *object* in subject focus conditions, the *subject* in object focus conditions), but not the focussed character (i.e., the *subject* in subject focus conditions, the *object* in object focus conditions). The fact that the focussed referent of the test sentence was not mentioned in the prior non-contrastive question follows from the function of the question to set up the following test sentence with focus on that previously unmentioned character—essentially, the question asks about something the speaker does not know, which is the focus of the answer. This is a reflection of real-world language use, where, as discussed in the Introduction, focussed constituents prototypically convey new information, while backgrounded constituents convey given information. Therefore we view the differences in number of character mentions for the non-contrastive question versus the test sentence as an inherent part of our manipulation of information structure. Note also that it could have been predicted that the referent with more previous mentions would be more accessible and therefore more likely to be considered as the referent of the pronoun (cf. the discussion on topic continuity above). However, our results showed more looks to the focussed referents, which had fewer previous mentions, suggesting that the imbalance in previous mentions at least did not drive our findings in any obvious way.

Even though adults displayed a higher magnitude of subject preference than children in *the broad focus* condition, it is still a surprising finding that children’s preference for subject looks relative to chance developed earlier (600 ms versus 1,100 ms past the pronoun onset). Adults displayed a small in magnitude but significant initial object and second mention preference in looks (until 750 ms); whereas children did not. An initial object preference is fairly common for standard SVO sentences, typically attributed to the recency effect (Hartshorne et al., 2015). This is unlikely the only reason in our study because it was designed to avoid recency effects by placing a location at the end of the SVO sentence (Järvikivi et al., 2005; e.g., *in front of the tree*). Another reason might be that the *broad focus* condition differed from the others because it asked *do you know what?*, rather than *do you know who?* This context is less suggestive that the next sentences are going to focus on characters, and if this contextual information is used more by adults then it would explain their relative delay.

Our working memory and language knowledge measures both accounted for developmental patterns in the processing of pronouns. One matter for future studies is to include a more comprehensive battery of tasks to measure these constructs, particularly to gain a more fine-grained understanding of the processing skills involved (Language Reading Research Consortium, 2015). The specific measure of working memory was chosen because it incorporates a storage and processing component, can be performed by 3-year-olds, and has a low semantic content so is less strongly related to language processing than tasks like verbal working memory span (Daneman and Merikle, 1996). Nevertheless, future work with older children would afford more complex span tasks (e.g., Gathercole et al., 2004), to examine if predictability holds across the specific conditions reported by the present study. For that same purpose, additional measures of language knowledge that test language exposure (Montag and MacDonald, 2015; Arnold et al., 2019) and grammatical knowledge could be included (Language Reading Research Consortium, 2015). This is especially important because there has been a lack of consistent effects for the role of individual differences in the general domain of children’s sentence comprehension, with some positive findings (Boyle et al., 2013; Blything et al., 2015; Qi et al., 2020), and others negative (de Ruiter et al., 2018, 2020). Further, the role of attentional effects and the overlap between pronoun interpretation and other language interpretive processes reflected in eye movements/fixations should be further explored in future work.

In summary, the present study demonstrated that, for both adults and young children, linguistic focus cues provided in a felicitous discourse context guide attention and modulate online processing of a pronoun. This was a robust finding for adults, as their baseline subject preference looks were enhanced when the subject was prosodically marked, and hindered when the object was prosodically marked. Both these findings extended to children who achieved medium to high scores in memory and language tasks. For the offline pronoun interpretation, both adults and children appeared to so robustly rely on other established cues like subjectivity and first mention that there was no clear influence of focus, either via prosody or clefts. Nevertheless, focus marking was attended to in online processing, reflecting the fact that several processes happen while listeners construct their discourse representation, of which pronoun resolution is just one part. For children, the additional presence of clefts seemed to in fact hinder their use of prosodic cues in subject focus conditions, whereas object clefts triggered an object preference, likely explainable as a first-mention effect—which was also reflected in offline responses by both children and adults. The incorporation of clefts in the experimental design allowed us to partially disentangle the effects of subjecthood and first mention, which normally converge in transitive English sentences. Comparing children and adults with the same experimental materials has highlighted that the misalignment of these cues, where it occurs, poses an even greater challenge for children. Finally, our study highlights the importance of investigating pronoun resolution in a felicitous context. While our results confirmed that focus affects online pronoun processing, they also showed that the final interpretation of the referential relations in the dialogues was not affected by focus, but driven by the constraints pertaining to the particular pronoun and the mental representation of a coherent discourse.

## Data Availability Statement

The datasets presented in this study can be found at the following link: https://osf.io/hw9re/.

## Ethics Statement

The studies involving human participants were reviewed and approved by the Research Ethics Board 2 of the University of Alberta. Written informed consent to participate in this study was provided by the participants’ legal guardian/next of kin.

## Author Contributions

AA, AT, and JJ designed the study and developed the materials. AT and LB oversaw data collection. LB conducted statistical analysis and wrote the first draft of the article. All authors contributed to editing the article and approved the submitted version.

## Conflict of Interest

The authors declare that the research was conducted in the absence of any commercial or financial relationships that could be construed as a potential conflict of interest.

## Publisher’s Note

All claims expressed in this article are solely those of the authors and do not necessarily represent those of their affiliated organizations, or those of the publisher, the editors and the reviewers. Any product that may be evaluated in this article, or claim that may be made by its manufacturer, is not guaranteed or endorsed by the publisher.

## References

[B1] Allen S. E. M. Hughes M. Skarabela B. (2015). “The role of cognitive accessibility in children’s referential choice,” in *The Acquisition of Reference. Trends in Language Acquisition Research*, eds Serratrice L. Allen S. E. M. (Amsterdam: John Benjamins), 1–24.

[B2] Ambridge B. Theakston A. Lieven E. Tomasello M. (2006). The distributed learning effect for children’s acquisition of an abstract grammatical construction. *Cogn. Dev.* 21 174–193. 10.1016/j.cogdev.2005.09.003

[B3] Aravind A. Freedman E. Hackl M. Wexler K. (2016). “Subject-object asymmetries in the acquisition of clefts,” in *Proceedings of the 40th Boston University Conference on Language Development (BUCLD 40)*, eds Scott J. Waughtal D. (Cascadilla Press), 1–17.

[B4] Armstrong M. E. Andreu L. Esteve-Gibert N. Prieto P. (2016). Children’s processing of morphosyntactic and prosodic cues in overriding context-based hypotheses: An eyetracking study. *Probus* 28 57–90. 10.1515/probus-2016-0004

[B5] Arnhold A. (2021). Prosodic focus marking in clefts and syntactically unmarked equivalents: Prosody – syntax trade-off or additive effects? *J. Acoust. Soc. Am.* 149 1390–1399. 10.1121/10.0003594 33765786

[B6] Arnhold A. Chen A. Järvikivi J. (2016). Acquiring complex focus marking: Finnish four- to ?ve-year-olds use prosody and word order in interaction. *Front. Psychol.* 7:1886. 10.3389/fpsyg.2016.01886 27990130 PMC5131328

[B7] Arnold J. E. (2001). The effect of thematic roles on pronoun use and frequency of reference continuation. *Discourse Processes* 31 137–162. 10.1207/S15326950DP3102_02

[B8] Arnold J. E. (2008). THE BACON not the bacon: How children and adults understand accented and unaccented noun phrases. *Cognition* 108 69–99. 10.1016/j.cognition.2008.01.001 18358460 PMC2518042

[B9] Arnold J. E. (2010). How speakers refer: the role of accessibility. *Lang. Ling. Compass* 4 187–203. 10.1111/j.1749-818X.2010.00193.x

[B10] Arnold J. E. Brown-Schmidt S. Trueswell J. (2007). Children’s use of gender and order-of-mention during pronoun comprehension. *Lang. Cogn. Proces.* 22 527–565. 10.1080/01690960600845950

[B11] Arnold J. E. Castro-Schilo L. Zerkle S. Rao L. (2019). Print exposure predicts pronoun comprehension strategies in children. *J. Child Lang.* 46 863–893. 10.1017/S0305000919000102 31124429

[B12] Arnold J. E. Eisenband J. G. Brown-Schmidt S. Trueswell J. C. (2000). The rapid use of gender information: Eyetracking evidence of the time-course of pronoun resolution. *Cognition* 76 B13–B26. 10.1016/S0010-0277(00)00073-1 10822045

[B13] Baayen R. H. (2008). *Analyzing linguistic data.* New York, NY: Cambridge University Press, 10.1017/CBO9780511801686

[B14] Baayen R. H. Davidson D. J. Bates D. M. (2008). Mixed–effects modelling with crossed random effects for subjects and items. *J. Memory Lang.* 59 390–412. 10.1016/j.jml.2007.12.005

[B15] Barr D. J. (2008). Analyzing ‘visual world’ eyetracking data using multilevel logistic regression. *J. Memory Lang.* 59 457–474. 10.1016/j.jml.2007.09.002

[B16] Barr D. J. Levy R. Scheepers C. Tily H. J. (2013). Random effects structure for confirmatory hypothesis testing: Keep it maximal. *J. Memory Lang.* 68 255–278. 10.1016/j.jml.2012.11.001 24403724 PMC3881361

[B17] Bates D. M. Maechler M. Bolker B. (2014). *lme4: Linear mixed–effects models using S4 classes*. R package version 0.999999–0.

[B18] Birch S. L. Garnsey S. M. (1995). The effect of focus on memory for words in sentences. *J. Memory Lang.* 34 232–267. 10.1006/jmla.1995.1011

[B19] Blewitt P. Rump K. M. Shealy S. E. Cook S. A. (2009). Shared Book Reading: When and how questions affect young children’s word learning. *J. Educ. Psychol.* 101 294–304. 10.1037/a0013844

[B20] Blything L. Davies R. Cain K. (2015). Young children’s comprehension of temporal relations in complex sentences: The influence of memory on performance. *Child Dev.* 86 1922–1934. 10.1111/cdev.12412 26309072 PMC4975597

[B21] Blything L. Iraola Azpiroy M. Allen S. Hert R. Järvikivi J. (2021). The influence of prominence cues in 7- to 10-year-olds’ pronoun resolution: Disentangling order of mention, grammatical role, and semantic role. *J. Child Lang.* 1–29. 10.1017/S0305000921000349 34167602

[B22] Boyle W. Lindell A. K. Kidd E. (2013). Investigating the role of verbal working memory in young children’s sentence comprehension. *Lang. Learn.* 63 211–242. 10.1111/lang.12003

[B23] Breen M. (2014). Empirical investigations of the role of implicit prosody in sentence processing. *Linguis. Lang. Compass* 8 37–50. 10.1111/lnc3.12061

[B24] Carreiras M. Gernsbacher M. A. Villa V. (1995). The advantage of first mention in Spanish. *Psychon. Bull. Rev.* 2 124–129. 10.3758/BF03214418 24203596 PMC4301433

[B25] Chafe W. L. (1976). “Givenness, contrastiveness, definiteness, subjects, and topics and point of view,” in *Subject and topic*, ed. Li C. (New York, NY: Academic Press), 27–55.

[B26] Colonna S. Hemforth B. (2014). “Information structure and pronoun resolution in German and French: Evidence from the visual-world paradigm,” in *Psycholinguistic approaches to meaning and understanding across languages*, eds Hemforth B. Schmiedtovà B. Fabricius-Hansen C. (Munich: Springer), 175–195. 10.1007/978-3-319-05675-3_7

[B27] Colonna S. Schimke S. Hemforth B. (2012). Information structure effects on anaphora resolution in German and French: A crosslinguistic study of pronoun resolution. *Linguistics* 50 991–1013. 10.1515/ling-2012-0031

[B28] Colonna S. Schimke S. Hemforth B. (2015). Different effects of focus in intra- and inter-sentential pronoun resolution in German. *Lang. Cogn. Neurosci.* 30 1306–1325. 10.1080/23273798.2015.1066510

[B29] Cooper-Cunningham R. Charest M. Porretta V. Järvikivi J. (2020). When couches have eyes: The effect of visual context on children’s reference processing. *Front. Comm.* 5:99. 10.3389/fcomm.2020.576236

[B30] Cowles H. W. Walenski M. Kluender R. (2007). Linguistic and cognitive prominence in anaphor resolution: Topic, contrastive focus and pronouns. *Topoi* 26 3–18. 10.1007/s11245-006-9004-6

[B31] Crawley R. Stevenson R. Kleinman D. (1990). The use of heuristic strategies in the interpretation of pronouns. *J. Psycholing. Res.* 4:245. 10.1007/BF01077259 2231480

[B32] Cutler A. Fodor J. A. (1979). Semantic focus and sentence comprehension. *Cognition* 7 49–59. 10.1016/0010-0277(79)90010-6 436402

[B33] Cutler A. Foss D. J. (1977). On the role of sentence stress in sentence processing. *Lang. Speech* 20 1–10. 10.1177/002383097702000101 592948

[B34] Daneman M. Merikle P. M. (1996). Working memory and language comprehension: A meta-analysis. *Psychon. Bull. Rev.* 3 422–433. 10.3758/BF03214546 24213976

[B35] de la Fuente I. (2015). *Putting pronoun resolution in context: The role of syntax, semantics, and pragmatics in pronoun interpretation.* PhD thesis, France: Université Paris Diderot.

[B36] de Ruiter L. E. Lieven E. V. M. Brandt S. Theakston A. (2020). Interactions between givenness and clause order in children’s processing of complex sentences. *Cognition* 198:104130. 10.1016/j.cognition.2019.104130 32032906

[B37] de Ruiter L. E. Theakston A. Brandt S. Lieven E. (2018). Iconicity affects children’s comprehension of complex sentences: The role of semantics, clause order, input, and individual differences. *Cognition* 171 202–224. 10.1016/j.cognition.2017.10.015 29197241

[B38] Declerck R. (1988). *Studies in Copular Sentences, Clefts, and Pseudo-Clefts.* Leuven: Leuven University Press.

[B39] Destruel E. Donaldson B. (2017). Second language acquisition of pragmatic inferences: Evidence from the French c’est-cleft. *Appl. Psycholing.* 38 703–732. 10.1017/S0142716416000400

[B40] DeVeaugh-Geiss J. P. Zimmermann M. Onea E. Boell A. C. (2015). Contradicting (not-)at-issueness in exclusives and clefts: An empirical study. *Semant. Ling. Theory* 25:373. 10.3765/salt.v25i0.3054 26464298

[B41] Drenhaus H. Zimmermann M. Vasishth S. (2011). Exhaustiveness effects in clefts are not truth-functional. *J. Neuroling.* 24 320–337. 10.1016/j.jneuroling.2010.10.004

[B42] Foraker S. McElree B. (2007). The role of prominence in pronoun resolution: Active versus passive representations. *J. Memory Lang.* 56 357–383. 10.1016/j.jml.2006.07.004

[B43] Freepik (2017). Available from https://www.freepik.com (accessed November 01, 2017).

[B44] Gathercole S. E. Pickering S. J. Ambridge B. Wearing H. (2004). The structure of working memory from 4 to 15 years of age. *Dev. Psychol.* 40 177–190. 10.1037/0012-1649.40.2.177 14979759

[B45] Gernsbacher M. A. Hargreaves D. J. (1988). Accessing sentence participants: The advantage of first mention. *J. Memory Lang.* 27 699–717. 10.1016/0749-596X(88)90016-2 PMC4266409 25520541

[B46] Givón T. (1983). *Topic Continuity in Discourse.* Amsterdam: John Benjamins.

[B47] Goldberg A. (2006). “Constructions at Work: The Nature of Generalization in Language,” in *Attention, intentions, and the structure of discourse. Computational Linguistics*, Vol. 12 eds Grosz B. Sidner C. L. (Oxford: Oxford University Press), 175–204.

[B48] Goodrich Smith W. Hudson Kam C. L. (2012). Knowing ‘who she is’ based on ‘where she is’: The effect of co-speech gesture on pronoun comprehension. *Lang. Cogn.* 4 75–98. 10.1515/langcog-2012-0005

[B49] Goodrich Smith W. Hudson Kam C. L. (2015). Children’s use of gesture in ambiguous pronoun interpretation. *J. Child Lang.* 42 591–617. 10.1017/S0305000915000045 25698162 PMC4396442

[B50] Grosz B. Sidner C. L. (1986). Attention, intentions, and the structure of discourse. *Comput. Linguist.* 12, 175–204.

[B51] Grüter T. Takeda A. Rohde H. Schafer A. J. (2018). Intersentential coreference expectations reflect mental models of events. *Cognition* 177 172–176. 10.1016/j.cognition.2018.04.015 29704855

[B52] Gundel J. K. Hedberg N. Zacharski R. (1993). Cognitive status and the form of referring expressions in discourse. *Language* 69 274–307. 10.2307/416535

[B53] Hadley E. B. Dickinson D. K. Hirsh-Pasek K. Colinkoff R. M. Nesbitt K. T. (2016). Examining the acquisition of vocabulary knowledge depth among preschool students. *Read. Res. Q.* 51 181–198. 10.1002/rrq.130

[B54] Hartshorne J. Nappa R. Snedeker J. (2015). Development of the first-mention bias. *J. Child Lang.* 42 423–446. 10.1017/S0305000914000075 24735525 PMC4451107

[B55] Hawthorne K. Arnhold A. Sullivan E. Järvikivi J. (2016). “Social cues modulate cognitive status of discourse referents,” in *Proceedings of the 38th Annual Conference of the Cognitive Science Society*, (Austin, TX: Cognitive Science Society), 562–567.

[B56] Hedberg N. (2000). The referential status of clefts. *Language* 76 891–920. 10.2307/417203

[B57] Hornby P. A. (1974). Surface structure and presupposition. *J. Verbal Learn. Verbal Behav.* 13 530–538. 10.1016/S0022-5371(74)80005-8

[B58] Huang Y. (2000). *Anaphora: A cross-linguistic approach.* Oxford: Oxford University Press.

[B59] Hughes D. Woodcock J. Funnell E. (2005). Conceptions of objects across categories: Childhood patterns resemble those of adults. *Br. J. Psychol.* 96 1–19. 10.1348/000712604X15446 15826321

[B60] Ito K. Speer S. R. (2008). Anticipatory effects of intonation: Eye movements during instructed visual search. *J. Memory Lang.* 58 541–573. 10.1016/j.jml.2007.06.013 19190719 PMC2361389

[B61] Ito K. Bibyk S. A. Wagner L. Speer S. R. (2014). Interpretation of contrastive pitch accent in six- to eleven-year-old English-speaking children (and adults). *J. Child Lang.* 41 84–110. 10.1017/S0305000912000554 23253142

[B62] Ito K. Jincho N. Minai U. Yamane N. Mazuka R. (2012). Intonation facilitates contrast resolution: Evidence from Japanese adults and 6-year olds. *J. Memory Lang.* 66 265–284. 10.1016/j.jml.2011.09.002

[B63] Järvikivi J. Pyykkönen-Klauck P. Schimke S. Colonna S. Hemforth B. (2014). Information structure cues for 4-year-olds and adults: Tracking eye movements to visually presented anaphoric referents. *Lang. Cogn. Neurosci.* 29 877–892. 10.1080/01690965.2013.804941

[B64] Järvikivi J. van Gompel R. P. G. Hyönä J. Bertram R. (2005). Ambiguous pronoun resolution: Contrasting the first-mention and subject-preference accounts. *Psycholog. Sci.* 16 260–264. 10.1111/j.0956-7976.2005.01525.x 15828971

[B65] Johnson-Laird P. N. (1983). *Mental models: Towards a cognitive science of language, inference and consciousness.* Cambridge, MA: Harvard University Press.

[B66] Kaiser E. (2011). Focusing on pronouns: Consequences of subjecthood, pronominalization and contrastive focus. *Lang. Cogn. Proc.* 26 1625–1666. 10.1080/01690965.2010.523082

[B67] Kaiser E. Trueswell J. (2008). Interpreting pronouns and demonstratives in Finnish: Evidence for a form-specific approach to reference resolution. *Lang. Cogn. Proc.* 23 709–748. 10.1080/0169096070

[B68] Káldi T. Babarczy A. (2021). Linguistic focus guides attention during the encoding and refreshing of Working Memory content. *J. Memory Lang.* 116:104187. 10.1016/j.jml.2020.104187

[B69] Keenan E. (1976). “Towards a universal definition of ‘Subject’,” in *Subject and Topic*, ed. Li C. (New York, NY: Academic Press), 61–77.

[B70] Kehler A. Kertz L. Rohde H. Elman J. L. (2008). Coherence and coreference revisited. *J. Semant.* 25 1–44. 10.1093/jos/ffm018 22923856 PMC3424618

[B71] Kember H. Choi J. Yu J. Cutler A. (2019). The processing of linguistic prominence. *Lang. Speech* 2019:80217. 10.1177/0023830919880217 31631754

[B72] Kidd E. (2013). The role of verbal working memory in children’s sentence comprehension. A critical review. *Topics Lang. Dis.* 33 208–223. 10.1097/TLD.0b013e31829d623e

[B73] Kidd E. Brandt S. Lieven E. Tomasello M. (2007). Object relatives made easy: A cross-linguistic comparison of the constraints influencing young children’s processing of relative clauses. *Lang Cogn. Proc.* 22 860–897. 10.1080/01690960601155284

[B74] Kiss K. É (1998). Identificational focus versus information focus. *Language* 74 245–273. 10.1353/lan.1998.0211

[B75] Kiss K. É (1999). “The English cleft construction as a focus phrase,” in *Boundaries of morphology and syntax*, ed. Mereu L. (Amsterdam: John Benjamins), 217–231. 10.1075/cilt.180.14kis

[B76] Koo T. K. Li M. Y. (2016). A guideline of selecting and reporting intraclass correlation coefficients for reliability research. *J. Chiropract. Med.* 15 155–163. 10.1016/j.jcm.2016.02.012 27330520 PMC4913118

[B77] Krifka M. (2007). “Basic notions of information structure,” in *Interdisciplinary studies of information structure 6, working papers of the SFB632*, eds Féry C. Fanselow G. Krifka M. (Potsdam: Universitatsverlag Potsdam), 13–56. 10.1093/oso/9780198814788.003.0002

[B78] Lambrecht K. (1994). *Information structure and sentence form. Topic, focus and the mental representations of discourse referents.* Cambridge, MA: Cambridge University Press.

[B79] Lambrecht K. (2001). A framework for the analysis of cleft constructions. *Linguistics* 39 463–516. 10.1515/ling.2001.021

[B80] Langlois V. J. Arnold J. E. (2020). Print exposure explains individual differences in using syntactic but not semantic cues for pronoun comprehension. *Cognition* 197:104155. 10.1016/j.cognition.2019.104155 31874414

[B81] Language Reading Research Consortium (2015). The dimensionality of language ability in young children. *Child Dev.* 86 1948–1965. 10.1111/cdev.12450 26509742

[B82] Linde C. (1979). “Focus of attention and the choice of pronouns in discourse,” in *Syntax and Semantics 12: Discourse and Syntax*, ed. Givón T. (London: Academic Press), 337–354. 10.1163/9789004368897_015

[B83] Maratsos M. P. (1973). The effects of stress on the understanding of pronominal coreference in children. *J Psycholing. Res.* 2 1–8. 10.1007/BF01067108 24197792

[B84] Matin E. Shao K. Boff K. (1993). Saccadic overhead: Information-processing time with and without saccades. *Percept. Psychophys.* 53 372–380. 10.3758/BF03206780 8483701

[B85] Montag J. L. MacDonald M. C. (2015). Text exposure predicts spoken production of complex sentences in 8- and 12-year-old children and adults. *J. Exp. Psychol. Gen.* 144 447–468. 10.1037/xge0000054 25844625 PMC4388064

[B86] Moore C. Harris L. Patriquin M. (1993). Lexical and prosodic cues in the comprehension of relative certainty. *J. Child Lang.* 20 153–167. 10.1017/S030500090000917X 8454680

[B87] Morrison C. M. Chappell A. W. Ellis P. T. (1992). Age of acquisition, not word frequency, affects object naming, not object recognition. *Memory Cogn.* 20 705–714. 10.3758/BF03202720 1435273

[B88] Niewland M. S. Van Berkum J. J. A. (2006). Individual differences and contextual bias in pronoun resolution: Evidence from ERPs. *Brain Research* 1118 155–167. 10.1016/j.brainres.2006.08.022 16956594

[B89] Patterson C. Esaulova Y. Felser C. (2017). The impact of focus on pronoun resolution in native and non-native sentence comprehension. *Second Lang. Res.* 33 403–429. 10.1177/0267658317697786

[B90] Paul R. (1985). The emergence of pragmatic comprehension: A study of children’s understanding of sentence-structure cues to given/new information. *J. Child Lang.* 12 161–179. 10.1017/S0305000900006292 3980600

[B91] Pinheiro J. C. Bates D. M. (2000). *Mixed–effects models in S and S–plus (statistics and computing*). New York, NY: Springer.

[B92] Porretta V. Kyröläinen A. van Rij J. Järvikivi J. (2018). “Visual world paradigm data: From preprocessing to nonlinear time-course analysis,” in *Intelligent Decision Technologies 2017. Proceedings of the 9th KES International Conference on Intelligent Decision Technologies (KES-IDT 2017)—Part II* (pp. 268–277), eds Czarnowski R. J. Howlett Jain L. C. (Cham: Springer International Publishing).

[B93] Prince E. (1981). “Toward a taxonomy of given-new information,” in *Radical Pragmatics*, ed. Cole P. (New York, NY: Academic Press), 223–256.

[B94] Psychology Software Tools (2018). *E-prime computer software for Nebraska.* Pittsburgh, PA: Psychology Software Tools.

[B95] Pyykkönen P. Järvikivi J. (2010). Activation and persistence of implicit causality information in spoken language comprehension. *Exp. Psychol.* 57 5–16. 10.1027/1618-3169/a000002 20176549

[B96] Pyykkönen P. Matthews D. Järvikivi J. (2010). Three-year-olds are sensitive to semantic prominence during online language comprehension: A visual world study of pronoun resolution. *Language and Cognitive Processes* 25 115–129. 10.1080/01690960902944014

[B97] Qi Z. Love J. Fisher C. Brown-Schmidt S. (2020). Referential context and executive functioning influence children’s resolution of syntactic ambiguity. *J. Exp. Psychol.* 2020:886. 10.1037/xlm0000886 32584080 PMC8287596

[B98] R Core Team. (2019). *R: A Language and Environment for Statistical Computing. Version 3.6.0.* Vienna: R Core Team.

[B99] Reinhart T. (1981). Pragmatics and linguistics: An analysis of sentence topics. *Philosophica* 27 53–94.

[B100] Sánchez-Alvarado C. (2020). “Syntactic and prosodic marking of subject focus in American English and Peninsular Spanish,” in *Hispanic Linguistics: Current issues and new directions*, eds Morales-Front A. Ferreira M. J. Leow R. P. Sanz C. (Amsterdam: John Benjamins Publishing Company), 184–203. 10.1075/ihll.26.09san

[B101] Schumacher P. Roberts L. Järvikivi J. (2017). Agentivity drives real-time pronoun resolution: Evidence from German er and der. *Lingua* 185 25–41. 10.1016/j.lingua.2016.07.004

[B102] Sekerina I. A. Trueswell J. C. (2012). Interactive processing of contrastive expressions by Russian children. *First Lang.* 32 63–87. 10.1177/0142723711403981 24465066 PMC3898858

[B103] Serratrice L. Allen S. E. M. (2015). “Introduction: an overview of the acquisition of reference,” in *The Acquisition of Reference. Trends in Language Acquisition Research*, eds Serratrice L. Allen S. E. M. (Amsterdam: John Benjamins), 1–24. 10.1075/tilar.15

[B104] Serratrice L. De Cat C. (2018). Individual differences in the production of referential expressions: The effect of language proficiency, language exposure and executive function in bilingual and monolingual children. *Lang. Cogn.* 23 371–386. 10.1017/S1366728918000962

[B105] Sheldon A. (1974). The role of parallel function in the acquisition of relative clauses in English. *J. Verbal Learn. Verb. Behav.* 13 272–281. 10.1016/S0022-5371(74)80064-2

[B106] Smyth R. (1994). Grammatical determinants of ambiguous pronoun resolution. *J. Psycholing. Res.* 23 197–229. 10.1007/BF02139085

[B107] Snow C. E. Cancino H. De Temple J. Schley S. (1991). “Giving formal definitions: A linguistic or metalinguistic skill?,” in *Language processing in bilingual children*, ed. Bialystock E. (Cambridge, UK: Cambridge University Press), 90–112. 10.1017/cbo9780511620652.007

[B108] Song H. Fisher C. (2005). Who’s “she”? Discourse prominence influences preschoolers’ comprehension of pronouns. *J. Memory Lang.* 52 29–57. 10.1016/j.jml.2004.06.012

[B109] Theakston A. (2012). “The spotty cow tickled the pig with a curly tail”: How do sentence position, preferred argument structure, and referential complexity affect children’s and adults’ choice of referring expression? *Appl. Psycholing.* 33 691–724. 10.1017/S0142716411000531

[B110] Theakston A. L. Coates A. Holler J. (2014). Handling agents and patients: Representational cospeech gestures help children comprehend complex syntactic constructions. *Dev. Psychol.* 50 1973–1984. 10.1037/a0036694 24773102

[B111] Trueswell J. C. Sekerina I. Hill N. Logrip M. (1999). The kindergarten-path effect: Studying on-line sentence processing in young children. *Cognition* 73 89–134. 10.1016/s0010-0277(99)00032-3 10580160

[B112] van Rij J. Hendriks P. van Rijn H. Baayen R. H. Wood S. N. (2019a). Analyzing the time course of pupillometric data. *Trends Hear. Sci.* 23 1–23. 10.1177/2331216519832483 31081486 PMC6535748

[B113] van Rij J. Vaci N. Wurm L. H. Feldman L. B. (2019b). “Alternative quantitative methods in psycholinguistics: Implications for theory and design,” in *Word knowledge and word usage: A cross-disciplinary guide to the mental lexicon*, eds Pirrelli V. I Dressler W. U. (Berlin: Mouton de Gruyter), 83–126. 10.1515/9783110440577-003

[B114] van Rij J. Wieling M. Baayen R. H. van Rijn H. (2020). *itsadug: Interpreting time series and autocorrelated data using GAMMs. R package version 2.3.*

[B115] van Valin R. D. Jr. LaPolla R. J. (1997). *Syntax: Structure, meaning, and function.* Cambridge, MA: Cambridge University Press.

[B116] Weighall A. R. Altmann G. T. M. (2010). The role of working memory and contextual constraints in children’s processing of relative clauses. *J. Child Lang.* 38 579–605. 10.1017/S0305000910000267 21040621

[B117] Wells B. Peppé S. Goulandris N. (2004). Intonation development from five to thirteen. *J. Child Lang.* 31 749–778. 10.1017/S030500090400652X 15658744

[B118] Wiebe S. A. Sheffield T. Nelson J. M. Clark C. A. C. Chevalier N. Espy K. A. (2011). The structure of executive function in 3-year-olds. *J. Exp. Child Psychol.* 108 436–452. 10.1016/j.jecp.2010.08.008 20884004 PMC3033982

[B119] Wood S. N. (2017). *mgcv: Mixed gam computation vehicle with automatic smoothness estimation*. R package. Available online at: https://cran.r-project.org/web/packages/mgcv (accessed June, 01, 2021).

[B120] Zhang L. Knoeferle P. (2012). “Visual context effects on thematic role assignment in children versus adults: Evidence from eye tracking in German,” in *Proceedings of the Annual Meeting of the Cognitive Science Society*, eds Miyake N. Peebles D. Cooper R. P. (Austin, TX: Cognitive Science Society), 2593–2598.

[B121] Zwaan R. A. Radvansky G. A. (1998). Situation models in language comprehension and memory. *Psycholog. Bull.* 123 162–185. 10.1037/0033-2909.123.2.162 9522683

