# Supplementary material for: The Influence of Focus Marking on Pronoun Resolution in Dialogue Context
Source: Front Psychol. 2021 Jul 26;12:684639. doi: 10.3389/fpsyg.2021.684639 (PMC8351791; doi:10.3389/fpsyg.2021.684639)
Supplement: Supplementary file 1 [file Table_1.docx]

Supplementary Material

# Inferential statistics for offline data

This section shows summary statistics of the Generalized Linear Mixed-effects Models (GLMMs) of offline pronoun attachment choice in adults (Supplementary Table 1) and children (Supplementary Table 2).

**Supplementary Table 1.** GLMM to show the main effect of condition on adult pronoun attachment choice (subject = 1; object =0).

| **Main model** | **M (*b*)** | | **SE** | **t** | | | **CI**  **2.5% 97.5%** | | | | |
| --- | --- | --- | --- | --- | --- | --- | --- | --- | --- | --- | --- |
| *Fixed effects:* |  |  | | |  | | |  | |  |  |
| (Intercept) | 2.83 | .34 | | | | 8.26 | | | 2.16 | 3.50 | |
| **Object focus-cleft present** | **-1.97** | **.29** | | | | **-6.68** | | **-2.54** | | **-1.39** | |
| Object focus-cleft absent | -.57 | .31 | | | | -1.83 | | -1.18 | | .04 | |
| Subject focus-cleft absent | .20 | .35 | | | | .58 | | -.48 | | .88 | |
| Subject focus-cleft present | -.03 | .33 | | | | -.09 | | -.68 | | .62 | |
| *Random effects:* |  |  | | | |  | | Variance | | SD | |
| Subject (intercept) |  |  | | | |  | | 1.37 | | 1.17 | |
| Item (intercept) |  |  | | | |  | | .47 | | .69 | |
| *Notes*: 1. Bold = predictor is significant at *p*<.05 or better, as indicated by CIs.  2. Number of observations = 1154; groups = 58 subjects and 20 items.  3. A positive coefficient indicates that the effect of differences between conditions was to increase the odds that a response would be the subject entity while a negative coefficient indicates that the effect of a factor was to decrease the odds that a object would be chosen. | | | | | | | | | | | |

**Supplementary Table 2.** GLMM to show the main effect of condition on children’s pronoun attachment choice (subject = 1; object =0).

| **Main model** | **M (*b*)** | | **SE** | ***t*** | | **CI**  **2.5% 97.5%** | | |
| --- | --- | --- | --- | --- | --- | --- | --- | --- |
| *Fixed effects:* |  |  | | |  | |  |  |
| (Intercept) | .74 | .25 | | | 2.90 | | .24 | 1.24 |
| **Object focus-cleft present** | **-.84** | **.29** | | | **-2.95** | | **-1.40** | **-.28** |
| Object focus-cleft absent | -.03 | .29 | | | -.11 | | -.59 | .53 |
| Subject focus-cleft absent | .36 | .29 | | | 1.21 | | -.22 | .93 |
| Subject focus-cleft present | .49 | .30 | | | 1.64 | | -.10 | 1.08 |
| *Random effects:* |  |  | | |  | | Variance | SD |
| Subject (intercept) |  |  | | |  | | .79 | .89 |
| *Notes*: 1. Bold = predictor is significant at *p*<.05 or better, as indicated by CIs  2. Number of observations = 632; groups = 37 subjects and 20 items  3. A positive coefficient indicates that the effect of differences between conditions was to increase the odds that a response would be the subject entity while a negative coefficient indicates that the effect of a factor was to decrease the odds that a subject would be chosen. | | | | | | | | |

# By-condition estimated effects plots for adult online data

This section provides larger sized, by-condition, summed plots with shaded confidence intervals for the main model of online processing in adults. These plots serve as evidence for when exactly each condition significantly differed from zero (i.e. a significant preference for the subject or object).


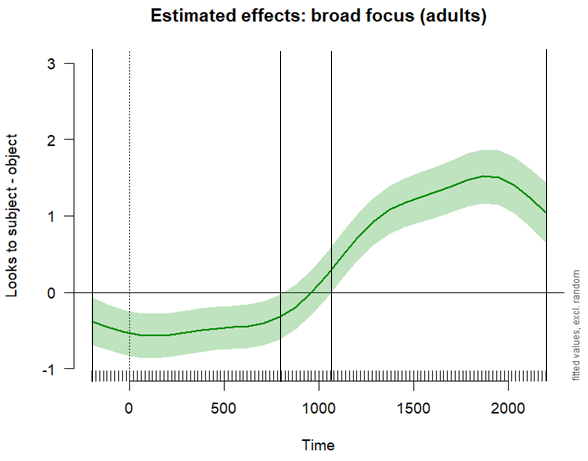


**Supplementary Figure 1.** Summed effects plot for the *broad focus* condition (adults) with vertical lines demonstrating when preferences differ from zero. A significant difference from zero is shown by white “daylight” between the 0 axis and shaded confidence intervals (marked here by vertical lines).

*
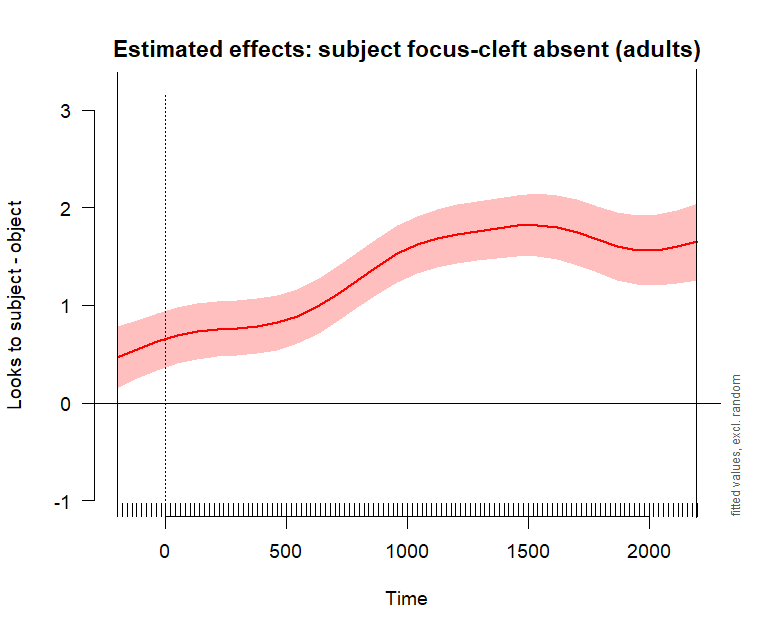
*

**Supplementary Figure 2.** Summed effects plot for the *subject focus-cleft absent* condition (adults) with vertical lines demonstrating when preferences differ from zero. A significant difference from zero is shown by white “daylight” between the 0 axis and shaded confidence intervals (marked here by vertical lines).

**
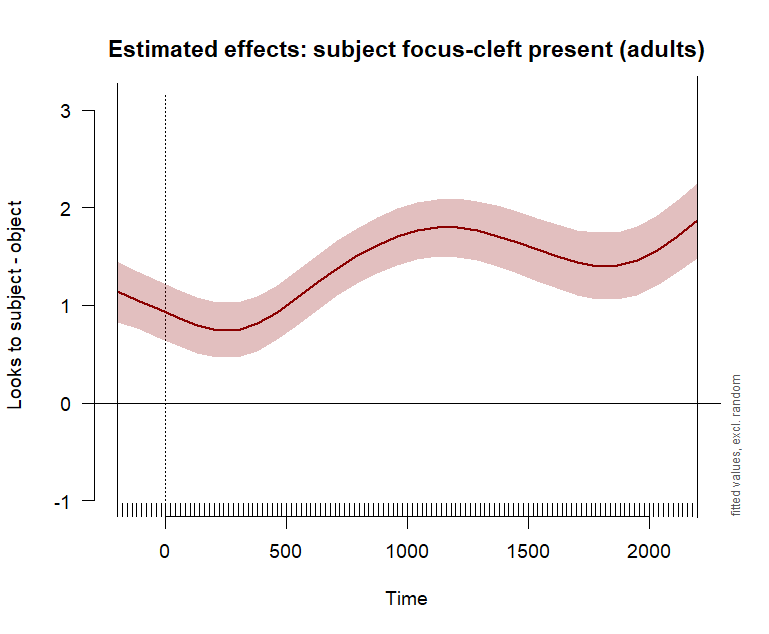
**

**Supplementary Figure 3.** Summed effects plot for the *subject focus-cleft present* condition (adults) with vertical lines demonstrating when preferences differ from zero. A significant difference from zero is shown by white “daylight” between the 0 axis and shaded confidence intervals (marked here by vertical lines).

**
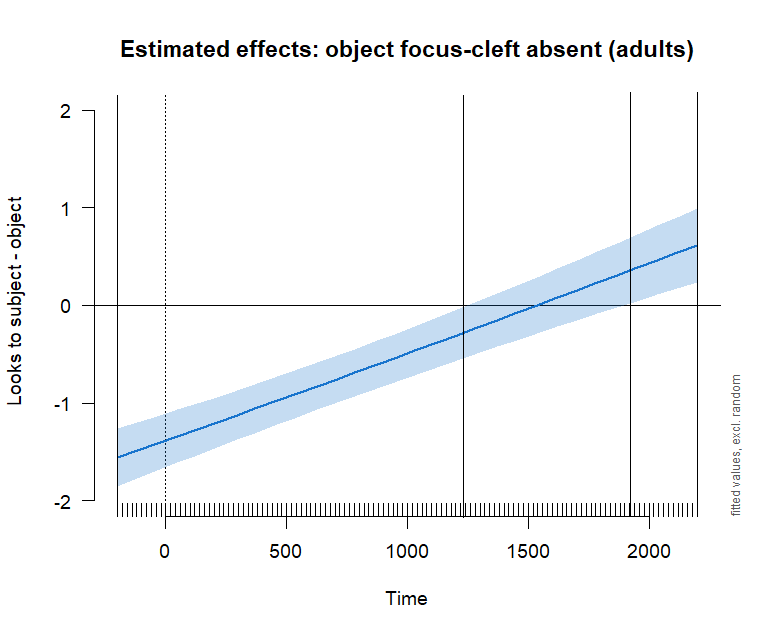
**

**Supplementary Figure 4.** Summed effects plot for the *object focus-cleft absent* condition (adults) with vertical lines demonstrating when preferences differ from zero. A significant difference from zero is shown by white “daylight” between the 0 axis and shaded confidence intervals (marked here by vertical lines).

**
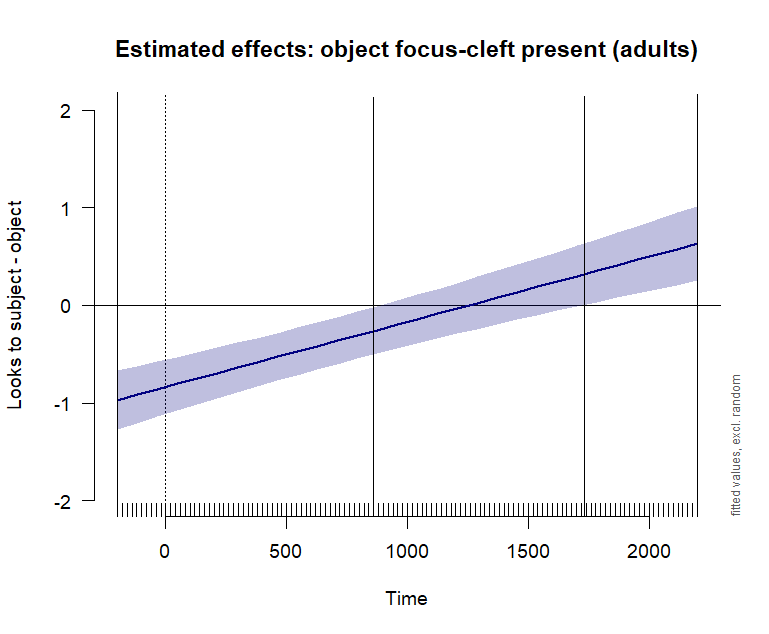
**

**Supplementary Figure 5.** Summed effects plot for the *object focus-cleft present* condition (adults) with vertical lines demonstrating when preferences differ from zero. A significant difference from zero is shown by white “daylight” between the 0 axis and shaded confidence intervals (marked here by vertical lines).

# Focus effects with and without cleft presence in adult online data

This section compares focus effects with and without the presence of cleft in addition to prosodic focus marking in the adult online data.

Supplementary Table 3 allows comparison between the two subject focus conditions by setting the *subject focus-cleft absent* condition as the reference level for the main model. It confirms the absence of a significant difference, as also seen in the left panel of Supplementary Figure 6. Similarly, relevelling to set the *object focus-cleft absent* condition as the reference level confirms that there is no significant difference between the object focus conditions (cf. Supplementary Table 4, also see right panel of Supplementary Figure 6).

**Supplementary Table 3.** Reference level = Subject focus-cleft absent (Dependent variable = subject – object). Summary statistics of the Generalized Additive Mixed Model for adults. Reporting parametric coefficients of sentence condition; and the smooth terms of sentence condition by time, with by-Subject and by-Item random smooths to time, and by-event random intercepts and slopes to time.

| **Parametric coefficients** |  | |  |  | |  |
| --- | --- | --- | --- | --- | --- | --- |
|  | | **Estimate** | **Std.Error** | **t** | **Pr(>\|t\|)** | |
| (Intercept) | | 1.30 | 0.25 | 5.19 | <0.01 | |
| **Broad focus** | | **-0.95** | **0.31** | **-3.05** | **<0.01** | |
| **Object focus-cleft present** | | **-1.47** | **0.31** | **-4.71** | **<0.01** | |
| **Object focus-cleft absent** | | **-1.78** | **0.31** | **-5.78** | **<0.01** | |
| Subject focus-cleft present | | 0.06 | 0.31 | 0.19 | 0.85 | |
| **Smooth terms** | |  |  |  |  | |
|  | | **edf** | **Ref.df** | **F** | **p-value** | |
| **s(Time): Subject focus-cleft absent** | | **5.20** | **6.50** | **2.81** | **<0.01** | |
| **s(Time): Broad focus** | | **6.36** | **7.65** | **6.48** | **<0.01** | |
| **s(Time): Object focus-cleft present** | | **1.01** | **1.02** | **10.97** | **<0.01** | |
| **s(Time): Object focus-cleft absent** | | **1.09** | **1.17** | **17.21** | **<0.01** | |
| **s(Time): Subject focus-cleft present** | | **5.92** | **7.24** | **3.45** | **<0.01** | |
| *Random effects* | | 253.81 | 521.00 | 2.14 | <0.01 | |
| s(Time,Subject) | | 75.94 | 179.00 | 2.61 | 0.01 | |
| s(Time,Item) | | 734.27 | 1095.00 | 6.75 | <0.01 | |
| s(Event) | | 628.54 | 1095.00 | 5.23 | <0.01 | |
| s(Time,Event) | | 5.20 | 6.50 | 2.81 | 0.01 | |

*Notes*. R-sq.(adj) = .49; Deviance explained = 49%; -ML = 180590; n = 133100


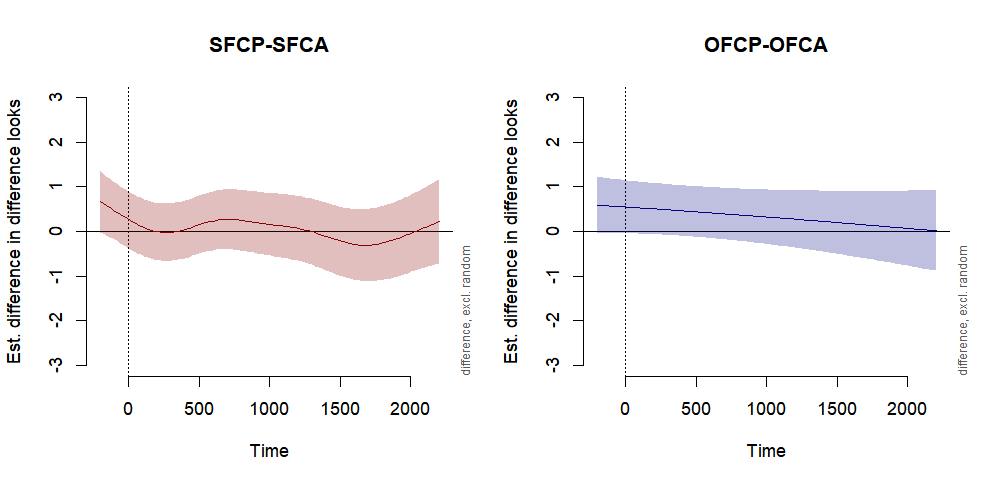


**Supplementary Figure 6.** Adults: Difference plots to confirm no significant difference within subject focus conditions (cleft present – cleft absent, left panel), or within object focus conditions (cleft present – cleft absent, right panel).

**Supplementary Table 4.** Reference level = Object focus-cleft absent (Dependent variable = subject – object). Summary statistics of the Generalized Additive Mixed Model for adults. Reporting parametric coefficients of sentence condition; and the smooth terms of sentence condition by time, with by-Subject and by-Item random smooths to time, and by-event random intercepts and slopes to time.

| **Parametric coefficients** |  | |  |  | |  |
| --- | --- | --- | --- | --- | --- | --- |
|  | | **Estimate** | **Std.Error** | **t** | **Pr(>\|t\|)** | |
| (Intercept) | | -0.48 | 0.24 | -1.98 | 0.05 | |
| **Broad focus** | | **0.83** | **0.31** | **2.70** | **0.01** | |
| Object focus-cleft present | | 0.32 | 0.31 | 1.03 | 0.31 | |
| **Subject focus-cleft present** | | **1.84** | **0.31** | **6.01** | **0.00** | |
| **Subject focus-cleft absent** | | **1.78** | **0.31** | **5.78** | **0.00** | |
| **Smooth terms** | |  |  |  |  | |
|  | | **edf** | **Ref.df** | **F** | **p-value** | |
| **s(Time): Object focus-cleft absent** | | **1.10** | **1.18** | **16.98** | **0.00** | |
| **s(Time): Broad focus** | | **6.36** | **7.65** | **6.48** | **0.00** | |
| **s(Time):Object focus-cleft present** | | **1.01** | **1.02** | **10.97** | **0.00** | |
| **s(Time):Subject focus-cleft present** | | **5.92** | **7.24** | **3.45** | **0.00** | |
| **s(Time):Subject focus-cleft absent** | | **5.17** | **6.47** | **2.80** | **0.01** | |
| *Random effects* | |  |  |  |  | |
| s(Time,Subject) | | 254.34 | 521.00 | 2.16 | 0.00 | |
| s(Time,Item) | | 75.93 | 179.00 | 2.61 | 0.01 | |
| s(Event) | | 733.81 | 1095.00 | 6.74 | 0.00 | |
| s(Time,Event) | | 628.38 | 1095.00 | 5.22 | 0.00 | |

*Notes*. R-sq.(adj) = .49; Deviance explained = 49%; -ML = 180590; n = 133100

# Subsidiary analysis on first-mention effect in adult online data

Supplementary Section 4 provides an analysis of the adult online data with first mention preference looks instead of subject preference looks as the dependent variable (looks to first mention minus looks to second mention). The reference level is object focus-cleft present. The *object focus-cleft present* condition is an exception to the other conditions, such that the object is fronted as the first mentioned entity (in all other conditions the object is second mention). Therefore this analysis enabled a check that the aforementioned effects were a combination of focus with grammatical role *and* order of mention, rather than only one of these factors: If the *object focus-cleft present* sentences do not differ in first mention preference relative to other conditions, it would indicate that the aforementioned subject preferences were driven by the combination of focus with order of mention alone, and not in combination with grammatical role.

Supplementary Table 5 shows the summary statistics, and Supplementary Figure 7 shows the difference plots.

Relative to the *object focus-cleft present* reference level, an enhanced first mention preference was displayed for *subject focus-cleft present* (650ms to 2200ms) and *subject focus-cleft absent* sentences (720ms to 2200ms). This indicates that the combination of focusing and grammatical role effects occur at time windows typical of pronoun effects in adults. Since those three conditions each focused the first mentioned entity, the *object focus-cleft-present* sentences differ from the other two only by focusing the object (rather than the subject). In other words, these results support the presence of a subject preference in addition to a first mention preference.

Conversely, the *object focus-cleft present* condition differs in nature to both *object focus-cleft absent* and *broad focus* sentences via its focusing of the first mentioned entity. Note that it additionally differs to *object focus-cleft absent* sentences via the cleft presence, and to *broad focus* via focus on the object, however the common difference was the focus on the first mention. *Object focus-cleft present* sentences were significantly more likely to show a preference for the first mention than the *object focus-cleft absent* condition (-200ms to 1180ms) and the *broad focus* condition (-200ms to 890ms), suggesting that the influence of focus on the first mention preference was effective immediately but also extends to when pronoun effects can apply (~400ms). However, later in the time course, *object focus-cleft present* sentences elicited a reduced first mention preference relative to *broad focus* (1300ms to 2200ms) and *object focus-cleft absent* sentences (1980ms to 2200ms), which additionally suggests that the influence of focus on first mention preferences did not persist as late into the time course. Broadly speaking, this analysis further supports that online looking preferences are weakened the more that focus, grammatical role and order of mention cues are put into conflict.

**Supplementary Table 5.** Reference level = Object focus-cleft present (Dependent variable = first – second mention). Summary statistics of the Generalized Additive Mixed Model for adults. Reporting parametric coefficients of sentence condition; and the smooth terms of sentence condition by time, with by-Subject and by-Item random smooths to time, and by-event random intercepts and slopes to time. For difference plots, see Figure 4 in the main text.

| **Parametric coefficients** |  | |  |  | |  |
| --- | --- | --- | --- | --- | --- | --- |
|  | | **Estimate** | **Std.Error** | ***t*** | **Pr(>\|t\|)** | |
| (Intercept) | | 0.20 | 0.24 | 0.82 | 0.41 | |
| Broad focus | | 0.01 | 0.31 | 0.03 | 0.97 | |
| **Object focus-cleft absent** | | **-0.84** | **0.31** | **-2.70** | **0.01** | |
| **Subject focus-cleft present** | | **1.02** | **0.31** | **3.30** | **<0.01** | |
| **Subject focus-cleft absent** | | **0.94** | **0.31** | **3.04** | **<0.01** | |
| **Smooth terms** | |  |  |  |  | |
|  | | **edf** | **Ref.df** | **F** | **p-value** | |
| **s(Time): Object focus-cleft present** | | **1.03** | **1.06** | **12.32** | **<0.01** | |
| **s(Time): Broad focus** | | **6.71** | **7.95** | **7.00** | **<0.01** | |
| **s(Time): Object focus-cleft absent** | | **2.28** | **2.91** | **7.88** | **<0.01** | |
| **s(Time):Subject focus-cleft present** | | **5.69** | **7.03** | **3.01** | **<0.01** | |
| **s(Time):Subject focus-cleft absent** | | **1.05** | **1.09** | **5.50** | **0.02** | |
| *Random effects* | |  |  |  |  | |
| s(Time,Subject) | | 246.67 | 521.00 | 1.94 | <0.01 | |
| s(Time,Item) | | 76.49 | 179.00 | 1.62 | 0.02 | |
| s(Event) | | 737.06 | 1095.00 | 6.52 | <0.01 | |
| s(Time,Event) | | 617.02 | 1095.00 | 4.88 | <0.01 | |

*Notes*. R-sq.(adj) = .48; Deviance explained = 49%; -ML = 180540; n = 133100


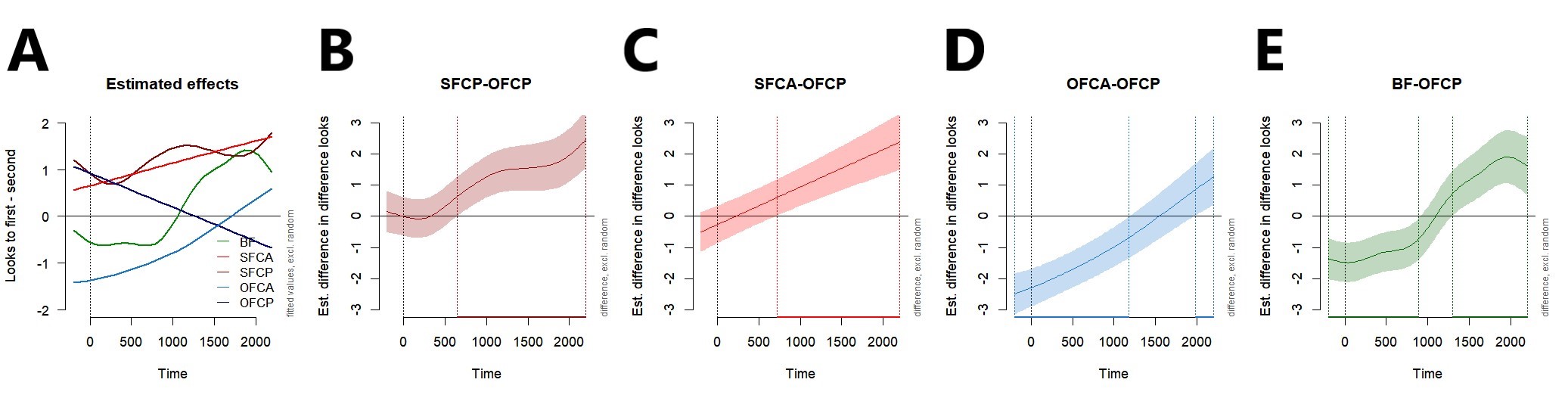


**Supplementary Figure 7.** Visualization of the summed effects derived from the “Subsidiary” models of adult (**A-E**) fixation patterns, with the random effects set to zero. Object focus-cleft present as reference; response variable = first mention – second mention. Left panel (**A**): Smooth terms for each time by condition term. Other panels (**B-E**): Difference plots visualizing the difference between each comparison condition with object focus-cleft present sentences. The difference plots are colored consistent with the grand means (Figure 2 of main manuscript) and smooth terms plot (left panels **A**), and have been abbreviated from left to right “SFCP” = *subject focus-cleft present* (dark red), “SFCA” = *subject focus-cleft absent* (red), “OFCA” = *object focus-cleft absent*, (blue), and “BF” = *broad focus* (green).

# By-condition estimated effects plots for children’s online data

This section provides larger sized individual summed plots for each condition that differed from zero according to the main model of online processing in children. Confidence intervals qualify the given significant time window.


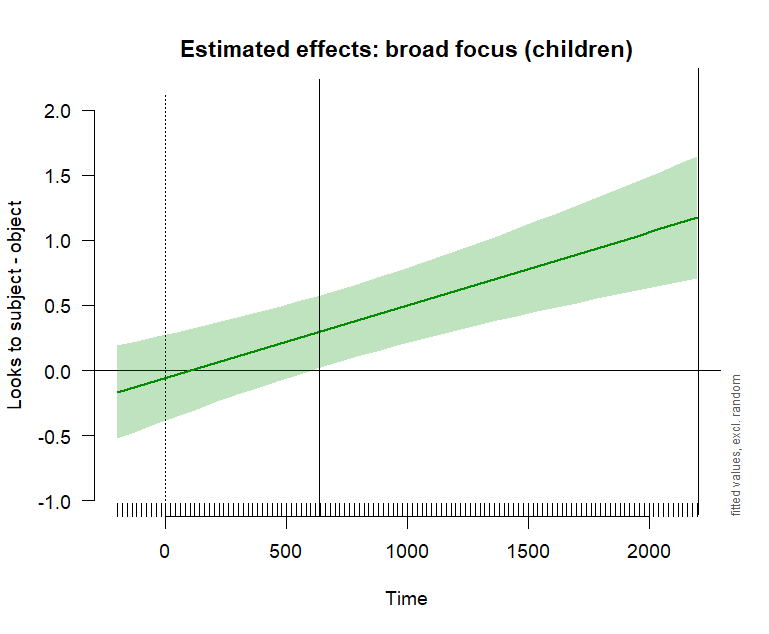


**Supplementary Figure 7.** Summed effects plot for the *broad focus* condition (children) with vertical lines demonstrating when preferences differ from zero. A significant difference from zero is shown by white “daylight” between the 0 axis and shaded confidence intervals (marked here by vertical lines).


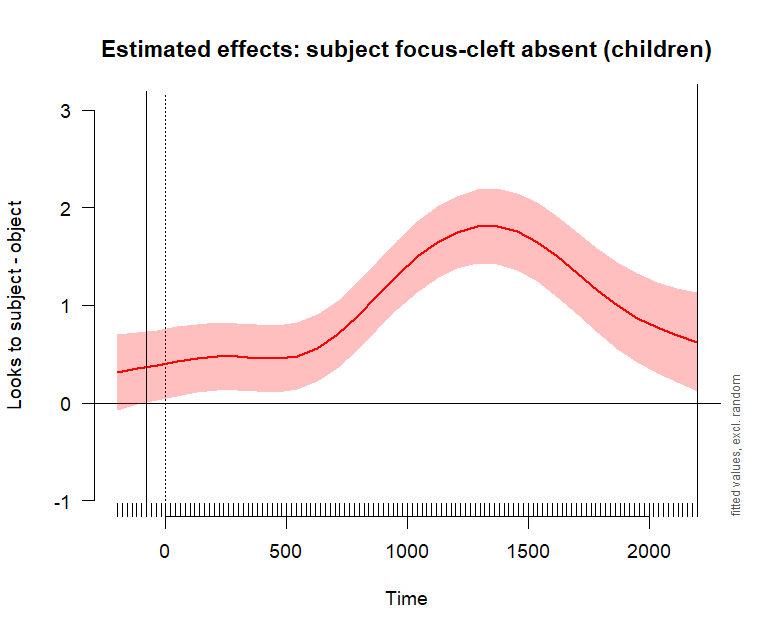


**Supplementary Figure 8.** Summed effects plot for the *subject focus-cleft absent* condition (children) with vertical lines demonstrating when preferences differ from zero. A significant difference from zero is shown by white “daylight” between the 0 axis and shaded confidence intervals (marked here by vertical lines).


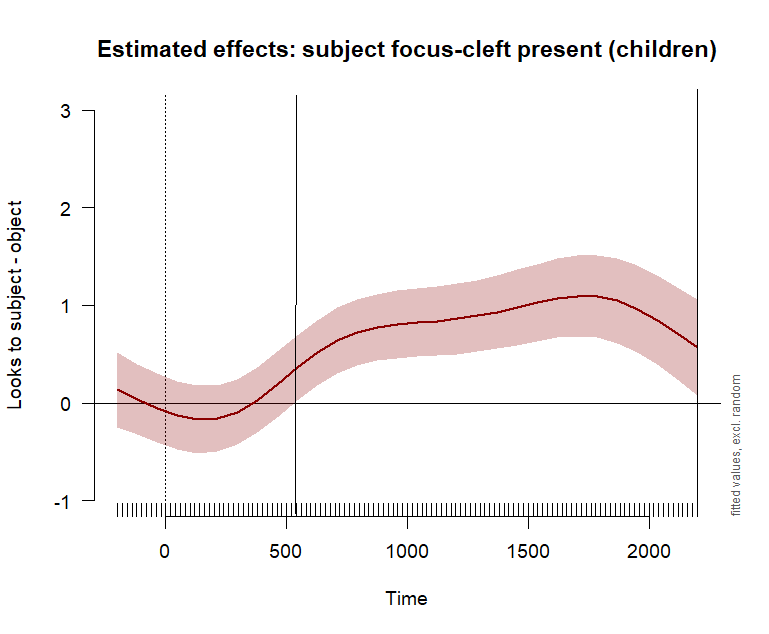


**Supplementary Figure 9.** Summed effects plot for the *subject focus-cleft present* condition (children) with vertical lines demonstrating when preferences differ from zero. A significant difference from zero is shown by white “daylight” between the 0 axis and shaded confidence intervals (marked here by vertical lines).

# Focus effects with and without cleft presence in children’s online data

This section compares focus effects with and without the presence of cleft in addition to prosodic focus marking in the children’s online data. Supplementary Table 6 shows summary statistics with the *subject focus-cleft absent* condition as the reference level for the main model. It confirms the absence of a significant difference between the subject focus conditions (also see the left panel of Supplementary Figure 10). Relevelling to set the *object focus-cleft absent* condition as the reference level shows a significant difference between the object focus conditions (cf. Supplementary Table 6, also see right panel of Supplementary Figure 10).

**Supplementary Table 6.** Reference level = Subject focus-cleft absent (Dependent variable = subject – object). Summary statistics of the Generalized Additive Mixed Model for children. Reporting parametric coefficients of sentence condition; and the smooth terms of sentence condition by time, with by-Subject and by-Item random smooths to time, and by-event random intercepts and slopes to time.

| **Parametric coefficients** |  | |  |  | |  |
| --- | --- | --- | --- | --- | --- | --- |
|  | | **Estimate** | **Std.Error** | **t** | **Pr(>\|t\|)** | |
| (Intercept) | | 0.97 | 0.31 | 3.14 | <0.01 | |
| Broad focus | | -0.47 | 0.39 | -1.20 | 0.23 | |
| **Object focus-cleft present** | | **-1.24** | **0.40** | **-3.08** | **<0.01** | |
| Object focus-cleft absent | | -0.57 | 0.40 | -1.43 | 0.15 | |
| Subject focus-cleft present | | -0.38 | 0.40 | -0.96 | 0.33 | |
| **Smooth terms** | |  |  |  |  | |
|  | | **edf** | **Ref.df** | **F** | **p-value** | |
| **s(Time): Subject focus-cleft absent** | | **5.67** | **7.02** | **4.56** | **<0.01** | |
| **s(Time):Broad focus** | | **1.06** | **1.10** | **4.25** | **0.03** | |
| s(Time): Object focus-cleft present | | 1.01 | 1.02 | 0.30 | 0.58 | |
| s(Time): Object focus-cleft absent | | 1.04 | 1.07 | 2.70 | 0.09 | |
| **s(Time): Subject focus-cleft present** | | **5.36** | **6.69** | **2.86** | **0.01** | |
| *Random effects* | |  |  |  |  | |
| s(Time,Subject) | | 155.31 | 323.00 | 2.97 | 0.00 | |
| s(Time,Item) | | 85.05 | 179.00 | 3.28 | 0.00 | |
| s(Event) | | 385.53 | 573.00 | 7.85 | 0.00 | |
| s(Time,Event) | | 351.84 | 573.00 | 6.87 | 0.00 | |

*Notes*. R-sq.(adj) = .48; Deviance explained = 48%; -ML = 83749 ; n = 69938


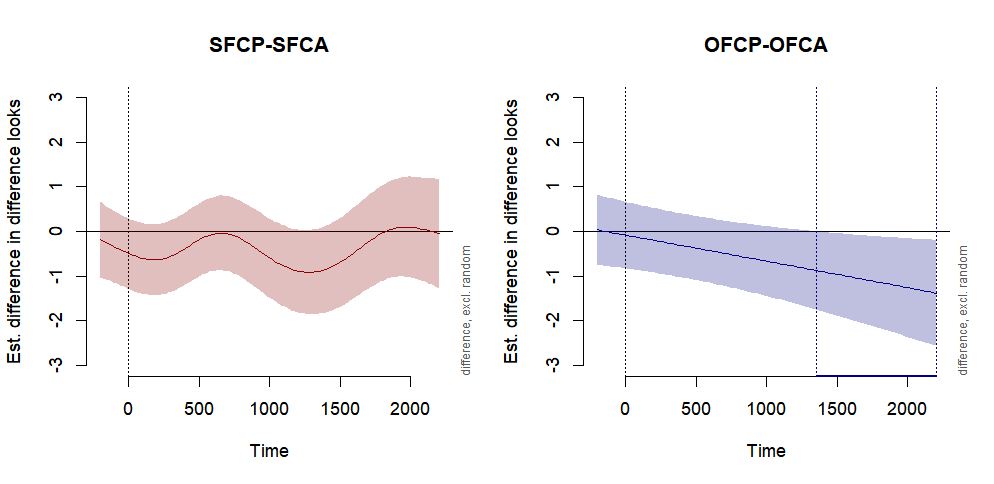


**Supplementary Figure 10.** Children: Difference plots to confirm no significant difference within subject focus conditions (cleft present – cleft absent, left panel). There was a significant difference within object focus conditions in the time window 1350ms– 2200ms (cleft present – cleft absent, right panel).

**Supplementary Table 7.** Reference level = Object focus-cleft absent (Dependent variable = subject – object). Summary statistics of the Generalized Additive Mixed Model for children. Reporting parametric coefficients of sentence condition; and the smooth terms of sentence condition by time, with by-Subject and by-Item random smooths to time, and by-event random intercepts and slopes to time.

| **Parametric coefficients** |  | |  |  | |  |
| --- | --- | --- | --- | --- | --- | --- |
|  | | **Estimate** | **Std.Error** | **t** | **Pr(>\|t\|)** | |
| (Intercept) | | 0.41 | 0.30 | 1.35 | 0.18 | |
| Broad focus | | 0.10 | 0.39 | 0.26 | 0.80 | |
| Object focus-cleft present | | -0.67 | 0.40 | -1.69 | 0.09 | |
| Subject focus-cleft present | | 0.18 | 0.39 | 0.47 | 0.64 | |
| Subject focus-cleft absent | | 0.57 | 0.40 | 1.43 | 0.15 | |
| **Smooth terms** | |  |  |  |  | |
|  | | **edf** | **Ref.df** | **F** | **p-value** | |
| s(Time): Object focus-cleft present | | 1.01 | 1.01 | 2.90 | 0.09 | |
| **s(Time): Broad focus** | | **1.07** | **1.13** | **4.11** | **0.03** | |
| s(Time): Object focus-cleft absent | | 1.03 | 1.06 | 0.28 | 0.60 | |
| **s(Time):Subject focus-cleft present** | | **5.36** | **6.70** | **2.86** | **0.01** | |
| **s(Time):Subject focus-cleft absent** | | **5.67** | **7.02** | **4.56** | **<0.01** | |
| *Random effects* | |  |  |  |  | |
| s(Time,Subject) | | 155.28 | 323.00 | 2.97 | <0.01 | |
| s(Time,Item) | | 84.54 | 179.00 | 3.13 | <0.01 | |
| s(Event) | | 385.99 | 573.00 | 7.86 | <0.01 | |
| s(Time,Event) | | 351.99 | 573.00 | 6.88 | <0.01 | |

*Notes*. R-sq.(adj) = .48; Deviance explained = 49%; -ML = 83749 ; n = 69938

# Subsidiary analysis on first-mention effect in children’s online data

The main model (3.2.4.1 of main manuscript) finding that, unlike adults, *object focus-cleft absent* (object = second mention) sentences did not significantly reduce children’s subject preference relative to the *broad focus* condition, suggests that the significant difference between *broad focus* and *object focus-cleft present* (object = first mention) sentences was more driven by a first mention preference than focusing of the object per se. This was confirmed in this section’s subsidiary analysis.

Supplementary Table 8 shows the summary statistics for an analysis of the children’s online data with first mention preference looks instead of subject preference looks as the dependent variable (looks to first mention minus looks to second mention). The reference level is object focus-cleft present.

Supplementary Figure 11:**E** shows that, in contrast to the main model (3.2.4.1 of main manuscript), the difference plot revealed no significant difference between the *broad focus* and *object focus-cleft present*. Thus, whereas the *subject* preference detected in the main model (relative to the *broad focus* condition) was significantly influenced by focus on the first mention and object, this subsidiary analysis revealed that *first mention* preference was not significantly influenced by a focus on the first mention and object. This indicates that the main model effect of *object focus-cleft present* was indeed driven by the fronting of the object. In this subsidiary model, *object focus-cleft present* was set as the reference so that it could be further compared to subject focus conditions. In the *subject focus-cleft absent* condition, subject (and first mention) focus enhanced the first mention preference relative to *object focus-cleft present* (Supplementary Figure 11**C**: 890ms to 1594ms). No significant differences were found in comparison to the *subject focus-cleft present or object focus-cleft absent* conditions Supplementary Figure 11**B and D**). Collectively, this further supports that preferences were strongest when focus is fully aligned onto the subject *and* first mention, but not in the additional presence of a cleft.

**Supplementary Table 8.** Reference level = Object focus-cleft present (Dependent variable = first – second mention). Summary statistics of the Generalized Additive Mixed Model for children. Reporting parametric coefficients of sentence condition; and the smooth terms of sentence condition by time, with by-Subject and by-Item random smooths to time, and by-event random intercepts and slopes to time.

| **Parametric coefficients** |  | |  |  | |  |
| --- | --- | --- | --- | --- | --- | --- |
|  | | **Estimate** | **Std.Error** | **t** | **Pr(>\|t\|)** | |
| (Intercept) | | 0.38 | 0.31 | 1.24 | 0.22 | |
| Broad focus | | 0.13 | 0.40 | 0.33 | 0.74 | |
| Object focus-cleft absent | | 0.04 | 0.41 | 0.09 | 0.93 | |
| Subject focus-cleft present | | 0.22 | 0.41 | 0.52 | 0.60 | |
| Subject focus-cleft absent | | 0.58 | 0.42 | 1.40 | 0.16 | |
| **Smooth terms** | |  |  |  |  | |
|  | | **edf** | **Ref.df** | **F** | **p-value** | |
| s(Time): Object focus-cleft present | | 1.02 | 1.03 | 0.29 | 0.60 | |
| **s(Time): Broad focus** | | **1.02** | **1.03** | **4.89** | **0.03** | |
| s(Time): Object focus-cleft absent | | 1.02 | 1.03 | 2.68 | 0.10 | |
| **s(Time): Subject focus-cleft present** | | **6.61** | **7.87** | **4.31** | **<0.01** | |
| **s(Time): Subject focus-cleft absent** | | **5.51** | **6.85** | **4.92** | **<0.01** | |
| *Random effects* | |  |  |  |  | |
| s(Time,Subject) | | 147.67 | 323.00 | 2.18 | <0.01 | |
| s(Time,Item) | | 100.71 | 179.00 | 3.33 | <0.01 | |
| s(Event) | | 394.21 | 573.00 | 9.37 | <0.01 | |
| s(Time,Event) | | 365.74 | 573.00 | 8.52 | <0.01 | |

*Notes*. R-sq.(adj) = .48; Deviance explained = 49%; -ML = 83778 ; n = 69938


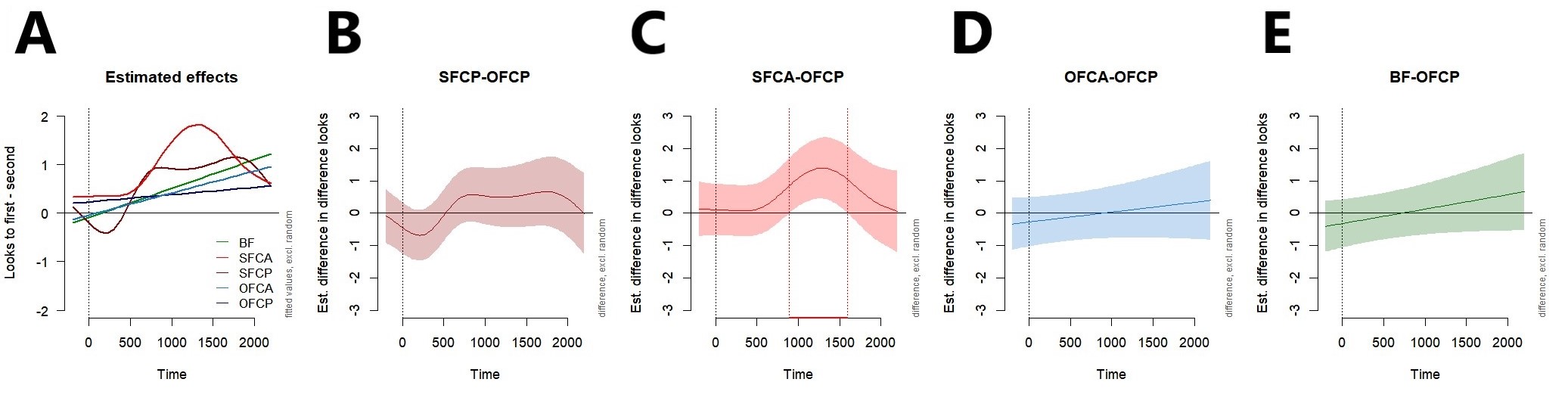


**Supplementary Figure 11.** Visualization of the summed effects derived from the “Subsidiary” models of 3- to 6-year old fixation patterns, with the random effects set to zero. Object focus-cleft present as reference; response variable = first mention – second mention. Left panel (**A**): Smooth terms for each time by condition term. Other panels (**B-E**): Difference plots visualizing the difference between each comparison condition with object focus-cleft present sentences. The difference plots are colored consistent with the grand means (Figure 2 of main manuscript) and smooth terms plot (left panels **A**), and have been abbreviated from left to right “SFCP” = *subject focus-cleft present* (dark red), “SFCA” = *subject focus-cleft absent* (red), “OFCA” = *object focus-cleft absent*, (blue), and “BF” = *broad focus* (green).

# Subsidiary analysis on first-mention effect for memory model of children’s online data

Supplementary Table 9 shows the summary statistics for the model of the children’s online data incorporating memory scores, with first mention preference looks as the dependent variable and *object focus-cleft present* as the reference level for condition.

**Supplementary Table 9.** Reference = Object focus-cleft present (Dependent variable = First – second mention). Summary statistics of the “Memory” Generalized Additive Mixed Model for 3- to 6-year-olds. Reporting parametric coefficients of sentence condition; and the smooth terms of each sentence condition by time, each sentence condition by memory, and a three-way interaction. Random effects of Time were by-Subject and by-Item random smooths, and by-event random intercepts and slopes.

| **Parametric coefficients** |  | |  | |  | |  | |
| --- | --- | --- | --- | --- | --- | --- | --- | --- |
|  | | **Estimate** | | **SE** | | ***t*** | | ***p*** |
| (Intercept) | | 0.53 | | 0.30 | | 1.74 | | 0.08 |
| **Broad focus** | | **-1.12** | | **0.42** | | **-2.68** | | **0.01** |
| Object focus-cleft absent | | -0.22 | | 0.41 | | -0.53 | | 0.60 |
| Subject focus-cleft present | | 0.06 | | 0.41 | | 0.14 | | 0.89 |
| Subject focus-cleft absent | | 0.53 | | 0.41 | | 1.29 | | 0.20 |
| **Smooth terms** | |  | |  | |  | |  |
|  | | **edf** | | **Ref.df** | | **F** | | ***p*** |
| s(Time): Object focus-cleft present | | 1.00 | | 1.01 | | 0.21 | | 0.65 |
| s(Time): Broad focus | | 1.00 | | 1.01 | | 1.91 | | 0.17 |
| s(Time):Object focus-cleft absent | | 1.00 | | 1.01 | | 2.70 | | 0.10 |
| **s(Time):Subject focus-cleft present** | | **5.80** | | **7.15** | | **2.58** | | **0.01** |
| **s(Time):Subject focus-cleft absent** | | **4.04** | | **5.17** | | **2.88** | | **0.01** |
| s(Memory): Object focus-cleft present | | 1.00 | | 1.00 | | 1.09 | | 0.30 |
| s(Memory): Broad focus | | 1.00 | | 1.00 | | 0.16 | | 0.69 |
| s(Memory):Object focus-cleft absent | | 1.00 | | 1.01 | | 1.26 | | 0.26 |
| s(Memory):Subject focus-cleft present | | 1.00 | | 1.00 | | 0.02 | | 0.89 |
| s(Memory):Subject focus-cleft absent | | 1.00 | | 1.00 | | 0.02 | | 0.89 |
| ti(Time, Memory): Object focus-cleft present | | 7.51 | | 9.70 | | 1.58 | | 0.10 |
| ti(Time, Memory): Broad focus | | 12.54 | | 14.32 | | 1.04 | | 0.30 |
| **ti(Time,** **Memory):Object focus-cleft absent** | | **8.10** | | **10.21** | | **2.25** | | **0.01** |
| ti(Time, Memory):Subject focus-cleft present | | 6.29 | | 8.31 | | 0.92 | | 0.56 |
| **ti(Time, Memory):Subject focus-cleft absent** | | **12.44** | | **14.34** | | **4.01** | | **<0.01** |
| *Random effects* | |  | |  | |  | |  |
| s(Time,Subject) | | 118.69 | | 268.00 | | 2.16 | | <0.01 |
| s(Time,Item) | | 95.31 | | 179.00 | | 2.96 | | <0.01 |
| s(Event) | | 329.64 | | 476.00 | | 9.46 | | <0.01 |
| s(Time,Event) | | 295.81 | | 476.00 | | 8.17 | | <0.01 |

*Notes*. R-sq.(adj) = .49; Deviance explained = 50%; -ML = 69410; n = 58806

# Vocabulary depth models of children’s online data

This section presents the results of analyzing children’s online data while incorporating the individual difference measure vocabulary depth.

Supplementary Table 10 shows the summary statistics for a model comparable to the memory model presented in Table 4 in the main text, with subject preference (looks to subject minus looks to object) as the dependent variable and *broad focus* as the reference level for condition. It contains a significant three-way interaction between time, vocabulary depths score and the *subject focus-cleft absent* condition, which is visualized in Supplementary Figure 11.

Supplementary Table 11 presents results of a subsidiary analysis with first mention preference (looks to first mention minus looks to second mention) as the dependent variable and *object focus-cleft present* as the reference level for condition, allowing for investigation of a first mention effect separate from a subject preference effect.

**Supplementary Table 10.** Reference = Broad focus (Dependent variable = subject – object). Summary statistics of the “Vocabulary depth” Generalized Additive Mixed Model for 3- to 6-year-olds. Reporting parametric coefficients of sentence condition; and the smooth terms of each sentence condition by time, each sentence condition by Vocabulary, and a three-way interaction. Random effects were by-Subject and by-Item random smooths to Time, and by-event random intercepts and slopes to Time.

| **Parametric coefficients** |  | |  | |  | |  | |
| --- | --- | --- | --- | --- | --- | --- | --- | --- |
|  | | **Estimate** | | **SE** | | **t** | | **p** |
| (Intercept) | | .51 | | .30 | | 1.68 | | .09 |
| Object focus-cleft present | | -.98 | | .41 | | -2.38 | | .02 |
| Object focus-cleft absent | | -.20 | | .41 | | -.49 | | .63 |
| Subject focus-cleft present | | -.03 | | .40 | | -.06 | | .95 |
| Subject focus-cleft absent | | .57 | | .41 | | 1.39 | | .17 |
| **Smooth terms** | |  | |  | |  | |  |
|  | | **edf** | | **Ref.df** | | **F** | | **p** |
| s(Time):Broad focus | | 1.01 | | 1.01 | | 2.27 | | .13 |
| s(Time):Object focus-cleft present | | 1.01 | | 1.01 | | .51 | | .48 |
| s(Time):Object focus-cleft absent | | 1.01 | | 1.02 | | 1.84 | | .17 |
| **s(Time):Subject focus-cleft present** | | **3.86** | | **4.96** | | **2.00** | | **.08** |
| **s(Time):Subject focus-cleft absent** | | **3.79** | | **4.87** | | **2.63** | | **.02** |
| s(Vocabulary):Broad focus | | 1.00 | | 1.00 | | .01 | | .94 |
| s(Vocabulary):Object focus-cleft present | | 1.00 | | 1.00 | | .24 | | .63 |
| s(Vocabulary):Object focus-cleft absent | | 1.00 | | 1.00 | | .23 | | .63 |
| s(Vocabulary):Subject focus-cleft present | | 1.00 | | 1.01 | | 1.33 | | .25 |
| s(Vocabulary):Subject focus-cleft absent | | 1.01 | | 1.01 | | .19 | | .67 |
| ti(Time,Vocabulary):Broad focus | | 1.06 | | 1.11 | | .28 | | .61 |
| ti(Time,Vocabulary):Object focus-cleft present | | 1.04 | | 1.08 | | .45 | | .51 |
| ti(Time,Vocabulary):Object focus-cleft absent | | 8.38 | | 1.74 | | 1.56 | | .11 |
| ti(Time,Vocabulary):Subject focus-cleft present | | 1.74 | | 2.18 | | 1.37 | | .27 |
| **ti(Time,Vocabulary):Subject focus-cleft absent** | | **1.09** | | **12.37** | | **3.46** | | **<.01** |
| *Random effects* | |  | |  | |  | |  |
| s(Time,Subject) | | 135.35 | | 277.00 | | 3.15 | | <.01 |
| s(Time,Item) | | 84.13 | | 179.00 | | 3.15 | | <.01 |
| s(Event) | | 331.22 | | 49.00 | | 8.16 | | <.01 |
| s(Time,Event) | | 298.70 | | 49.00 | | 7.06 | | <.01 |

Notes. R-sq.(adj) = .49; Deviance explained = 49%; -ML = 71351; n = 6050. This model used a square root transformation of the vocabulary depth predictor because it was more normally distributed – however, the non-transformed version revealed the same pattern of significant findings.

*
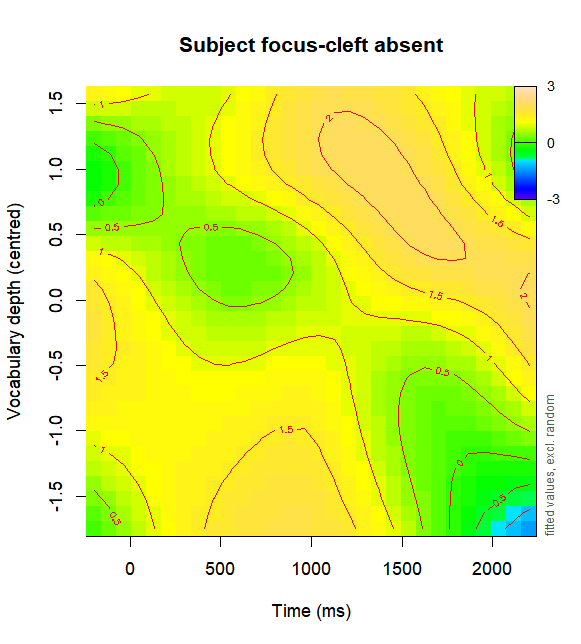
*

**Supplementary Figure 12.** Children: Contour plot of three-way interactions between Time (x-axis), Vocabulary scores on the vocabulary depth task (y-axis) and subject focus-cleft absent. *Notes:* Yellow indicates a subject preference, whereas green indicates a reduced subject preference. Blue colors represent object preference, but these are too minor to interpret.

**Supplementary Table 11.** Reference = Object focus-cleft present (Dependent variable = first – second mention). Summary statistics of the “Vocabulary depth” Generalized Additive Mixed Model for 3- to 6-year-olds. Reporting parametric coefficients of sentence condition; and the smooth terms of each sentence condition by time, each sentence condition by vocabulary, and a three-way interaction. Random effects were by-Subject and by-Item random smooths to Time, and by-event random intercepts and slopes to Time.

| **Parametric coefficients** |  | |  | |  | |  | |
| --- | --- | --- | --- | --- | --- | --- | --- | --- |
|  | | **Estimate** | | **SE** | | **t** | | ***p*** |
| (Intercept) | | 0.54 | | 0.32 | | 1.69 | | 0.09 |
| Broad focus | | <0.01 | | 0.42 | | -0.01 | | 1.00 |
| Object focus-cleft absent | | -0.17 | | 0.43 | | -0.41 | | 0.68 |
| Subject focus-cleft present | | -0.03 | | 0.43 | | -0.07 | | 0.94 |
| Subject focus-cleft absent | | 0.54 | | 0.44 | | 1.24 | | 0.22 |
| **Smooth terms** | |  | |  | |  | |  |
|  | | **edf** | | **Ref.df** | | **F** | | ***p*** |
| s(Time): Object focus-cleft present | | 1.02 | | 1.04 | | 0.24 | | 0.64 |
| s(Time): Broad focus | | 1.01 | | 1.01 | | 2.61 | | 0.11 |
| s(Time):Object focus-cleft absent | | 1.01 | | 1.01 | | 2.19 | | 0.14 |
| **s(Time):Subject focus-cleft present** | | **5.92** | | **7.26** | | **2.71** | | **0.01** |
| **s(Time):Subject focus-cleft absent** | | **3.55** | | **4.57** | | **2.67** | | **0.02** |
| s(Vocabulary): Object focus-cleft present | | 1.00 | | 1.00 | | 0.16 | | 0.69 |
| s(Vocabulary): Broad focus | | 1.00 | | 1.00 | | 0.09 | | 0.76 |
| s(Vocabulary):Object focus-cleft absent | | 1.00 | | 1.00 | | 0.26 | | 0.61 |
| s(Vocabulary):Subject focus-cleft present | | 1.00 | | 1.00 | | 1.55 | | 0.21 |
| s(Vocabulary):Subject focus-cleft absent | | 1.00 | | 1.00 | | 0.37 | | 0.55 |
| ti(Time,Vocabulary): Object focus-cleft present | | 1.02 | | 1.03 | | 2.87 | | 0.09 |
| ti(Time,Vocabulary): Broad focus | | 5.16 | | 6.97 | | 0.38 | | 0.92 |
| **ti(Time,Vocabulary):Object focus-cleft absent** | | **10.89** | | **13.11** | | **2.43** | | **<0.01** |
| ti(Time,Vocabulary):Subject focus-cleft present | | 1.37 | | 1.63 | | 0.28 | | 0.60 |
| **ti(Time,Vocabulary):Subject** **focus-cleft absent** | | **9.00** | | **11.30** | | **2.71** | | **<0.01** |
| *Random effects* | |  | |  | |  | |  |
| s(Time,Subject) | | 125.06 | | 277.00 | | 2.26 | | <0.01 |
| s(Time,Item) | | 91.70 | | 179.00 | | 2.90 | | <0.01 |
| s(Event) | | 340.98 | | 490.00 | | 9.56 | | <0.01 |
| s(Time,Event) | | 307.19 | | 490.00 | | 8.33 | | <0.01 |

*Notes*. R-sq.(adj) = .49; Deviance explained = 49%; -ML = 71375; n = 60500
